# PTX3 is expressed in terminal lymphatics and shapes their organization and function

**DOI:** 10.3389/fimmu.2024.1426869

**Published:** 2024-11-21

**Authors:** Andrea Doni, Marina Sironi, Annalisa Del Prete, Fabio Pasqualini, Sonia Valentino, Ivan Cuccovillo, Raffaella Parente, Michela Calvi, Antonella Tosoni, Gianluca Vago, Manuela Nebuloni, Cecilia Garlanda, Annunciata Vecchi, Barbara Bottazzi, Alberto Mantovani

**Affiliations:** ^1^ Unit of Multiscale and Nanostructural Imaging, IRCCS Humanitas Research Hospital, Milan, Italy; ^2^ Cellular and Humoral Innate Immunity Lab, IRCCS Humanitas Research Hospital, Milan, Italy; ^3^ Department of Molecular and Translational Medicine, University of Brescia, Brescia, Italy; ^4^ Clinical and Experimental Immunology Lab, IRCCS Humanitas Research Hospital, Milan, Italy; ^5^ Pathology Unit, L. Sacco Hospital, Department of Biomedical and Clinical Sciences, University of Milan, Milan, Italy; ^6^ Experimental Immunopathology Lab, IRCCS Humanitas Research Hospital, Milan, Italy; ^7^ Department of Biomedical Sciences, Humanitas University, Milan, Italy; ^8^ William Harvey Research Institute, Queen Mary University of London, London, United Kingdom

**Keywords:** innate immunity, pattern recognition molecule, extracellular matrix, tissue homeostasis, lymphatic system

## Abstract

**Introduction:**

The lymphatic system is a multifaceted regulator of tissue homeostasis and an integral part of immune responses. Previous studies had shown that subsets of lymphatic endothelial cells (LEC) express *PTX3*, an essential component of humoral innate immunity and tissue homeostasis.

**Methods:**

In the present study using whole-mount imaging and image-based morphometric quantifications, *Ptx3*-targeted mice and *in vivo* functional analysis, we investigated the involvement of PTX3 in shaping and function of the lymphatic vasculature.

**Results:**

We found that PTX3 is localized in the extracellular matrix (ECM) surrounding human and murine lymphatic vessels (LV). In murine tissues, PTX3 was localized in the ECM close to LV terminals and sprouting. *Ptx3*-deficient mice showed LV abnormalities in the colon submucosa and diaphragm, including a disorganized pattern and hyperplasia of initial LV capillaries associated with altered distribution of tight junction-associated molecules. Mice with LEC-restricted PTX3 gene inactivation showed morphological and organization abnormalities similar to those observed in *Ptx3*-deficient animals. Ptx3-deficient mice showed defective fluid drainage from footpads and defective dendritic cell (DC) trafficking.

**Discussion:**

Thus, PTX3 is strategically localized in the ECM of specialized LV, playing an essential role in their structural organization and immunological function.

## 1 Introduction

The main function of the lymphatic system consists in controlling the balance of body fluids, returning extravasated fluid through a system of increasingly large conduits, from initial (or terminal) lymphatic vessel (LV) capillaries and collecting LVs to lymph nodes (LNs) interposed to bloodstream (1). In the embryo, LVs develop as a result of the establishment of functional blood circulation, from which lymphatic endothelial cells (LECs) differentiate from blood endothelial cells (ECs) of the main veins and migrate into the adjacent mesenchyme to form the first primitive lymphatic structures (1). Expansion of the lymphatic vasculature occurs through sprouting of LVs or recruitment of progenitors to form initial capillaries, functional in absorbing interstitial fluid and site of lymph formation (1–3). Functional specialization of different clusters of LECs is associated with molecular signatures in single-cell transcriptomic studies (4–8).

The lymphatic system is an integral part of immunity (9–12). Functions related to immunological surveillance include recruitment, migration, and trafficking of immune cells through the release of LEC-derived chemokines (e.g., CCL21, CXCL9 and CXCL12, constitutively expressed by LECs, or CCL19 and CCL12 differentially expressed by LECs in inflammatory conditions) (13–15), LEC-restricted expression of the atypical receptor ACKR2 (16, 17), and transport of bacteria and foreign antigens to LNs and lymphoid structures (18–21). The lymphatic system actively contributes to the innate immune response by functioning as a barrier, and hence limiting the spread of pathogens, through phagocytosis by neutrophils or macrophages residing in the subcapsular sinus of LNs (22) or through a local active coagulation that engulfs pathogens in transiently occluded LVs (23). Abnormalities in LV function are implicated in inflammatory and immunological disorders, infections (22, 24), and cancer (25–27).

A mature lymphatic vasculature closely associated with lymphoid tissues consists of LECs joined by functionally specialized cell–cell junctions to maintain the integrity of LVs (28). Terminal capillaries consist of blind-ended LVs composed by a single, thin, non-fenestrated lymphatic LEC layer not surrounded by pericytes and smooth muscle cells (SMCs). The basement membrane is almost absent in the lymphatic capillaries (1) and LECs are closely interconnected with the surrounding extracellular matrix (ECM) (29–31) by elastic anchoring fibrils that extend into connective tissue, a close interaction that plays an important role in LV function (1–3). Initial LVs are highly permeable and drain interstitial fluid through discontinuous LEC junctions, distinct from the zipper-like junctions observed in collecting LVs, where VE-cadherin, a functional adhesive protein in establishing the normal integrity of the endothelial barrier, and other tight junction molecules such as occludin, claudin-5, ZO-1, and JAM-A intermittently localize (32, 33). These specialized junctions can allow fluid entry through openings between buttons. Collecting LVs are generally not tethered to ECM but contain an inner endothelium surrounded by a medial layer of circular SMC and thus may support a circumferential hoop stress. Collecting vessels contain one-way valves that aid in lymph propulsion and prevent lymph backflow. LVs within the skeletal muscle lack an SMC layer and do not exhibit intrinsic pumping. The increase in lymphatic flow from the skeletal muscle is probably governed by the direct action of the muscular fibers around LVs (28). The diaphragm has both LVs in muscle tissue capable of draining pleural fluids and LVs protruding into the tendon enveloped by mesothelium to drain the peritoneal fluid, as well as absorbing excess interstitial fluid within the skeletal muscle of the diaphragm itself (34). The latter plays an important role in defense against microbes in infections of the gastrointestinal or urogenital system (28, 34). Pleural and peritoneal LVs are interconnected but function separately; indeed, lymph formed on the pleural side does not appear in the network that originates on the peritoneal side of the muscle, and *vice versa*. LVs that protrude on the peritoneal side are flattened with large lumens, also called *lacunae* (28, 34).

Structural and biophysical properties of the ECM *per se* influence the formation and function of lymphatics (30, 31, 35, 36). An ECM interaction with the abluminal part of initial lymphatics via anchoring filaments underlies the formation and transport of lymph through LV expansion and compression (1–3), which is also regulated by a direct effect of the ECM in response to tissue stress, such as contraction of surrounding muscles (36). Evidence points to the relevance of ECM molecules in LV function in homeostasis and pathology (30, 31). Integrin- or non-integrin-mediated interactions of LECs with ECM constituents regulate lymphangiogenesis in organogenesis, wound healing, immune defense, and cancer (37). Hyaluronan (HA), a major glycosaminoglycan ECM component localized around LVs, acts as a pro-lymphangiogenic signal once processed as a fragment (19). LYVE-1 expressed by LECs is fundamental in HA turnover and functional interactions between LECs and ECM in inflammation (19). LVs themselves affect the ECM structure and composition. LEC-derived ADAMTS3 is involved in the proteolytic activation of VEGFC (38), whereas MMP-1 and MMP-2 directly regulate lymphangiogenesis promoting invasion through ECM remodeling (39).

The long pentraxin 3 (PTX3) is an essential component of the humoral innate immune system (40, 41), and a distant relative of the short pentraxins C-reactive protein and serum amyloid P component (41, 42). PTX3 is rapidly produced as a homo-octameric glycoprotein by immune and stromal cells, most prominently macrophages and ECs, in response to pro-inflammatory signals and infection. The role of PTX3 in humoral innate immunity (40, 43, 44) includes recognition of pathogens through interaction with microbial moieties and facilitation of opsonophagocytosis via FcγR (45), therefore acting as a functional ancestor of antibodies. Moreover, PTX3 acts as a natural FGF2 trap, inhibiting the lymphangiogenic activity exerted by a VEGF-A/FGF2/sphingosine-1-phosphate cocktail (46), and regulates inflammation via interaction with P-selectin (47) and orchestration of complement activation and regulation (48). PTX3 interacts with ECM components and plays a role in tissue homeostasis and repair (49–52). By multivalent contacts with TNF-stimulated gene 6 (TSG-6) and inter-alpha-trypsin inhibitor (IαI) heavy chains (HCs) (52), PTX3 regulates the organization of HA-HC-rich cumulus oophorous ECM, thus ensuring oocyte fertilization (51). By interacting with fibrin and plasminogen, PTX3 was shown to regulate the thrombotic response (53) and to promote fibrinolysis in wounds, hence favoring tissue repair (49, 54). Thus, the functions of PTX3, an evolutionary conserved molecule, are diverse, encompassing immunity, ECM remodeling, and tissue repair.

Early gene expression profiling analysis revealed that human (55, 56) and murine (57) LECs constitutively express *PTX3*. In single-cell transcriptomics, an immune-interacting subtype of LECs with high *Ptx3* expression was identified (8, 12, 58). In a genetic model of lymphatic malformations by oncogenic *PIK3CA* mutation, *Ptx3* expression was observed in abnormal LV sprouts associated with vascular lesions (58). A *Ptx3* ^+^ subtype (PTX3-LECs) included two groups of capillary nonproliferating and proliferating LECs with high expression of *Lyve-1* and transcripts encoding for regulators of innate and adaptive immune responses (8, 12, 58). In the same vein, *Ptx3* expression was reported to define a subpopulation of LN LECs characterized by high expression of genes involved in the regulation of lymphangiogenesis and immune responses (4, 8, 12, 59).

In spite of its essential role in immunity and expression in specialized LEC clusters, the localization and actual function of PTX3 in LV remain unknown. The present study was designed to investigate the localization and functional significance of PTX3 in the lymphatic system taking advantage of gene targeted mice.

## 2 Materials and methods

### 2.1 Mice

Procedures involving animal handling and care conformed to protocols approved by the Humanitas Clinical and Research Center in compliance with national (D.L. N.116, G.U., suppl. 40, 18-2-1992 and N.26, G.U. 4 March 2014) and international law and policies (EEC Council Directive 2010/63/EU, OJ L 276/33, 22-09-2010; National Institutes of Health Guide for the Care and Use of Laboratory Animals, US National Research Council, 2011). The study was approved by the Italian Ministry of Health (approval no. 29/2014 PR). Mice were housed in the specific pathogen-free animal facility of the Humanitas Clinical and Research Center in individually ventilated cages and were used between 8 and 12 weeks of age. All efforts were made to minimize the number of animals used and their suffering.

*Ptx3*-deficient mice were generated as previously described (40) and were used on a 129/SV or C57BL/6J inbred genetic background matched with the appropriate wild-type (WT) controls (Charles River Laboratories). *Tsg-6* (*Tnfaip6*)-deficient mice on 129/SV inbred genetic background were purchased from the Jackson Laboratory (JAX^®^ Mice, USA). Prof. Taijia Mäkinen (Department of Immunology, Genetics and Pathology, Vascular Biology, University of Uppsala, Sweden) developed (60) and provided *Prox1-CreER^T2^* mice on C57BL/6J genetic background. *Ptx3^flox/flox^* mice were developed by Oxgene (Oxford, UK) and bred with *Prox1-CreER^T2^* to generate *Prox1-CreER^T2^*/*Ptx3^flox/flox^* mice. Mice were maintained in heterozygous expression of *CreER^T2^*. To selectively induce deletion of the *lox-P*-flanked Cre sequence of murine *Ptx3*, Tamoxifen (#T5648; Sigma-Aldrich/Merck) dissolved in corn oil (#C8267; Sigma-Aldrich/Merck) at 20 mg/mL was administered i.p. (100 µL/mouse) for 5 days. LPS (2 µg/mouse; *E*. *coli* O111:B4; Sigma-Aldrich/Merck) was administered intraperitoneally (i.p.) 16 h before blood collection.

### 2.2 Bone marrow transplantation

C57BL/6J WT or *Ptx3^−/−^* were lethally irradiated with a total dose of 9 Gray (Gy) (EN 60601; RADGIL, Italy). Two hours later, mice were injected i.v. with 5 × 10^6^ nucleated bone marrow (BM) cells obtained by flushing of the cavity of freshly dissected femur from a WT or *Ptx3^−/−^* donors. Recipient mice received gentamycin (0.4 mg/mL in drinking water) starting at day 10 before irradiation to day 15 after. Chimeric mice were used for morphometric evaluations 8 weeks after BM transplantation.

### 2.3 Immunostaining and light microscopy

For immunohistochemistry (IHC), the formalin-fixed paraffin-embedded (FFPE) human tissues (collection of anonymized tissues donated for research purposes, Pathology Unit, L. Sacco Hospital, Milan) were cut at 5 µm, deparaffinized, hydrated, and unmasked with EDTA 0.25 mM (pH 8.0). For immunofluorescence analysis, frozen tissues were cut at 8 µm and fixed with 4% paraformaldehyde (PFA). Endogenous peroxidases were blocked for 20 min with Peroxidazed 1 (BioCare Medical, USA) and unspecific binding sites were blocked with PBS containing calcium and magnesium (PBS^2+^) (pH 7.4) + 1% bovine serum albumin (BSA) (#9048-46-8; Sigma-Aldrich/Merck) + 0.02% NP-40 (#9016-45-9; Merck Millipore) (blocking buffer) for 30 min. An affinity-purified rabbit polyclonal anti-human PTX3 (0.25 µg/mL), a monoclonal mouse anti-D2/40 (1:100; #MCA2543; AbD Serotec), and a monoclonal mouse anti-FVIII/Von Willebrand Factor (1:200; #M0616; Dako Cytomation) diluted in blocking buffer were used. In IHC, MACH4 Universal HRP-Polymer (#M4U534L; BioCare Medical, USA) was used as secondary antibody. Reaction was developed with Betazoid DAB (#BDB2004L; BioCare Medical, USA) and counterstained with hematoxylin and eosin (H&E). For immunofluorescence, goat anti-rabbit Alexa Fluor^®^ 488 (both at 1 µg/mL) and goat anti-mouse Alexa Fluor^®^ 647 (both at 1 µg/mL) (Thermo Fisher Scientific–Invitrogen Molecular Probes, USA) were used. DAPI was used for the nuclear staining. Slides were mounted with ProLong Gold antifade reagent (Invitrogen-Molecular Probes, USA).

Whole mount staining protocols were repurposed as per the previous description (32). Briefly, the vasculature of anesthetized (ketamine 100 mg/kg, xylazine, 10 mg/kg; i.p.) mice was perfused for 2 min with 1% PFA in PBS^2+^ (pH 7.4) from a canula inserted through the left ventricle under a constant pressure of 120 mmHg, applied using a hand-help pump with an attached manometer. Diaphragm, trachea, colon, and ear skin were removed, immersed in 1% PFA solution, and incubated overnight (o/n) at 4°C protected from light and under constant rotation. Tissues were washed with PBS^2+^ (pH 7.4) + 1% BSA and stained immunohistochemically by incubating whole mounts with the primary antibodies diluted in PBS^2+^ (pH 7.4) containing 0.5% Triton X-100 (#T8787), 2% BSA, 5% normal goat (#D9663) or donkey (#G9023) serum (all from Sigma-Aldrich/Merck), 0.01% glycine, and 0.001% sodium azide (blocking buffer). The following reagents were used: rabbit polyclonal anti-LYVE-1, 1:1,000 (2 µg/mL; #ab14917, Abcam); Armenian hamster monoclonal anti-mouse CD31 (PECAM-1) (2 µg/mL; #2H8; Chemicon International Inc., USA) or rat monoclonal anti-mouse CD31 (0.5 µg/mL; #MEC 13.3, BD Biosciences); goat polyclonal anti-mouse PTX3 (5 µg/mL; #AF2166; R&D Systems); goat polyclonal anti-mouse TSG-6 (5 µg/mL; #AF2326; R&D Systems); rat monoclonal anti-mouse CD144 (VE-cadherin) (1 µg/mL; IgG_1_; #eBioBV13; eBiosciences); and rat monoclonal anti-mouse F4/80 (1 µg/mL; #eBioBV13; eBiosciences). The corresponding irrelevant IgGs were used as control: IgGs from normal donkey and goat serum were prepared in-house by purification on Protein G-Sepharose™ Fast Flow (GE Healthcare, Sweden); rat IgG_2A_ (#R35-95) and IgG_1_ (#R3-34) (from BD Biosciences); and Armenian hamster IgG (#HTK888; BioLegend). Species-specific secondary antibodies labeled with Alexa^®^ Fluor 488, 594, and 647 (Thermo Fisher Scientific–Invitrogen Molecular Probes, USA) or Cy5 (Jackson ImmunoResearch Laboratories Inc.) were used at 2 µg/mL. All immunostainings were performed o/n at 4°C on a platform rocker. After each step, tissues were washed five times (total washing time, 4–6 h), each time by adding PBS^2+^ (pH 7.4) containing 0.5% Triton X-100 and 0.05% Tween-20. The same protocol and the following antibodies were used for staining the cell subpopulations in the tissues: FITC-conjugated rat anti-Ly6G (2 µg/mL; #1A8; BD Biosciences), Brillant Violet^™^ (BV421)-conjugated rat anti-CD11b (1 µg/mL; #M1/70; BD Biosciences), and APC-conjugated hamster anti-CD11c (2 µg/mL; #HL3; BD Biosciences). In the same experiment, the rabbit polyclonal anti-LYVE-1 (2 µg/mL; #ab14917; Abcam) and a donkey anti-rabbit IgG (H+L) Alexa^®^ Fluor 546 (1:1,000, 2 µg/mL; #A10040; Thermo Fisher Scientific–Invitrogen Molecular Probes, USA) were used. Samples were mounted in antifade mounting medium on glass slides stored at 4°C and protected from light. The tissue sides facing up were the serosa of colon, the peritoneal surface of diaphragm, and tracheal mucosa. Ear skin was divided into the two flaps and observed in its inner part. To strengthen evidence related to the penetrance of the phenotypes, imaging analyses of PTX3 expression and morphometric measurements were conducted in mice with either 129/SV or C57BL/6J genetic background.

Confocal microscopy analysis was performed on mounted organs using a confocal FV1000 Olympus microscope operating in sequential channel mode at 488-, 543-, and 635-nm excitations. The resulting fluorescence emission was collected using a 500- to 550-nm (for Alexa^®^ Fluor 488), a 565- to 615-nm (for Alexa^®^ Fluor 546), and a 655- to 750-nm (for Alexa^®^ Fluor 647, Cy5 and APC) band-pass filter. Samples were imaged with an Olympus UPLXAPO 10× (NA 0.4) or with an Olympus UPLXAPO 60× oil (NA 1.3) lens in optical sections of 0.75 μm (10×) or 0.3 µm (60×). Part of the samples were also analyzed in a sequential scanning mode with a Leica TCS SP8 confocal microscope and an immersion lens HC PL APO 20× (NA 0.75) equipped with Leica Application Suite X software (LASX; version 3.5.5.19976). BV421, FITC, Alexa Fluor^®^ 546, and APC were excited with a 405-nm diode UV laser, an argon laser at 488 nm, and a white laser at 546 and 650 nm, and emission was collected from 415–450 nm (BV421), 490–550 nm (FITC), 555–620 nm (Alexa Fluor^®^ 546), and 650–750 nm (APC), respectively. The *z*-stack images (1,024 × 1,024 pixels) were acquired at a resolution of 1 Airy unit to allow 3D reconstruction. Acquisitions were also performed in a sequential scanning mode using a Leica TCS SP8 confocal microscope at Airy Unit 1 with an HC PL APO 10× (NA 0.45) lens equipped with Leica Application Suite X software (LASX; version 3.5.5.19976). Images were obtained after *z*-stack acquisition using the same parameters (0.75 μm/slice; 1,024 × 1,024 pixels). Image deconvolution was performed by Huygens Professional software (Scientific Volume Imaging B. V.; version 19.10) and presented as medium intensity projection (MIP).

### 2.4 Image analysis

The confocal *z*-stack RGB images were converted using the Imaris File Converter (version X64 9.7.2; Bitplane), imported to Imaris software (version X64 9.7.2; Bitplane) to 3D reconstruct imaged organs. Images were assembled and voxels with thresholded fluorescence intensities were assigned for each color channel. Isosurfaces were rendered from these voxels and smoothed with a Gaussian filter, creating 3D reconstructions in which the spatial resolution was conserved. The quantification of PTX3^+^ volume around the LVs was performed after threshold of the positive signal on the basis of the background signal obtained in *Ptx3*-deficient mice and IgG isotype controls and presented as the sum of PTX3^+^ voxels per mm^3^ of tissue. The LYVE-1^+^ LV area and volume were measured after isosurface rendering of confocal *z*-stack images and expressed as µm^2^ (area) and µm^3^ (volume). Vessel length (µm) was measured after drawing by Filament Tracer mode by Imaris software. The reported morphometric values are the mean of the indicated number of regions per mouse tissue each of 1.65, 1.20, and 0.32 mm^2^, and expressed as mm^2^ of tissue. The number of terminal LVs was measured as stereological point counting and expressed per mm^2^. As also previously reported (61), the number of CD144^+^ (VE-cadherin)-stained zipper and button junctions was counted after *z*-stack confocal acquisition using an Olympus UPLXAPO 60× oil (NA 1.3) lens in optical sections of 0.3 µm/slice in 3D reconstructed images (1,024 × 1,024 pixels). Since the distinction of the terminal LVs in the colonic submucosa is unequivocal, this organ was used for quantifications of zipper-to-button junctions in WT vs. *Ptx3^−/−^* mice. *N* = 3 acquisitions each representing two to four terminal LVs were considered for analysis in four (WT) or five (*Ptx3^−/−^*) animals and expressed as percentage of button (discontinuity of the junctional expression of CD144) junctions on total LYVE-1^+^ LECs. Tissue leucocytes were measured in the vicinity of the LVs after 3D reconstruction of the *z*-stack (0.75 μm/slice; 1,024 × 1,024 pixels) of the entire organ and using the Spot Detection module of the Imaris software (version X64 9.7.2; Bitplane). The fluorescence signal corresponding to each channel was automatically thresholded on the basis of intensity quality and diameter size (10 ± 2 µm) parameters to identify each individual cell as a spot with its own ID. The spots corresponding to the reconstruction of all individual cells in each tissue volume were counted automatically and expressed as the sum per mm^2^ of tissue area. Results report counts of LYVE-1^+^ CD11b^+^ macrophages (colon and skin), Ly6G^-^ CD11b^+^ mono-macrophages (diaphragm), Ly6G^+^ CD11b^+^ neutrophils (colon, diaphragm, and skin), and CD11c^+^ DCs (colon, diaphragm, and skin).

### 2.5 Electron microscopy

For electron microscopy (EM) examination, mouse (129/SV genetic background) diaphragms were fixed in buffered 2.5% glutaraldehyde solution, post-fixed in 1% osmium tetroxide solution, and embedded in epoxy resin (Durcupan ACM Fluka). Sections were stained with 3% uranyl acetate and Reynold’s lead citrate solution and examined using the transmission electron microscope EM109 Zeiss. LVs were distinct from blood vessels on the base of their ultrastructural specific features, including discontinuous basal membrane and anchoring fibrils directly departing from the endothelial plasma membrane (62). Initial LVs were identified by the presence of the peculiar primary valve structures. Sub-mesothelial initial LVs were recognized on the base of the characteristic mesothelial–lymphatic *stomata* structure (62). Micrographs at ×9,000, ×21,000, and ×36,000 magnification of LVs from wt and *Ptx3^−/−^* mice were compared. Peri-*stomata* areas were analyzed on micrographs at ×9,000 magnification.

### 2.6 *In vivo* draining of solutes

To obtain information on the physiological tissue homeostasis in WT and *Ptx3*-deficient animals (129/SV), the ability to drain Evans Blue (EB) dye was evaluated. Briefly, a 10% solution of EB dye (#314-13-6; Sigma-Aldrich/Merck) in saline was injected (10 µL/mice) into the footpads of hind limbs of anesthetized (ketamine 100 mg/kg, xylazine, 10 mg/kg; i.p.) mice, as already described (63). Draining local and distal (popliteal and iliac) LNs were harvested at different time points (15, 30, and 60 min) after injection. EB dye was extracted by incubation of the LNs in *N*,*N*-dimethylformamide (#68-12-2; Sigma-Aldrich/Merck) o/n at 55°C and quantified spectrophotometrically as absorbance measured at *A*_620nm_.

### 2.7 *In vivo* trafficking of DCs

A skin painting model was used with fluorescein isothiocyanate isomer (FITC) (#3326-32-7; Sigma-Aldrich/Merck) dissolved in a 50:50 (vol/vol) acetone–dibutyl phthalate (BDH) mixture just before application. Mice were painted on the shaved abdomen with 200 µL of 5 mg/mL FITC. After 6 and 12 h, inguinal LNs were individually collected and disaggregated with collagenase–DNase I blend (#B20221; Sigma-Aldrich/Merck) for 30 min. Total cells obtained from each LN were counted in Turk’s solution and then stained with phycoerythrin (PE) anti-CD11c (#HL3, IgG_1_; BD Biosciences) and analyzed by a FACS LSRFortessa™ Flow Cytometer (BD Biosciences). The number of double-positive DCs per LN was calculated based on the frequency of FITC^+^CD11c^+^ cells evaluated by FACS. As stated in figure legends, experiments were conducted in mice with both 129/SV and C57BL/6J genetic background.

### 2.8 *Salmonella* infection

The ΔaroA auxotrophic variant of the *Salmonella enterica* serovar Typhimurium strain used in this study shows an attenuated ability to replicate *in vivo* (64). Bacteria were grown at 37°C in Luria broth with 100 µg/mL of ampicillin (all from Sigma-Aldrich/Merck). A total of 10^9^ bacteria in 200 µL of 5% NaHCO_3_ were orally administered (per os) to WT and *Ptx3^−/−^* mice on C57BL/6J background. After 24 h, mesenteric LNs were aseptically removed and incubated for 2 h at 37°C with 50 µg/mL gentamycin to kill external bacteria. Organs were then digested with 1 mg/mL collagenase-D (Sigma-Aldrich/Merck) for 30 min at 37°C, lysed with 0.5% sodium-deoxycholate (Sigma-Aldrich/Merck), and plated on 1.5% agar plates in Luria broth. After o/n culture, CFU were evaluated, and results were reported as CFU/10^6^ plated cells.

### 2.9 ELISA

As previously described (49), Numc-Immuno™ 96-well plates (Sigma-Aldrich/Merck) were coated o/n with 100 ng/50 µL of a purified Emilin-1 fragment (N-terminal gC1q domain, aa 676 ≈ 1,016; #EPR14678; AbCam) or native purified plasminogen (#528175; Sigma-Aldrich/Merck). Nonspecific binding to the plates was blocked by incubation for 2 h at 25°C with 2% BSA in PBS^2+^ before incubation (1 h at 25°C) with 100 µL of purified PTX3, C and N domain in PBS^2+^ (at indicated pHs) containing 0.05% (vol/vol) Tween-20. Recombinant human PTX3, C- and N-terminal domains were purified by immunoaffinity from culture supernatants as previously described (49). After washing, binding was detected by incubation with 100 µL of biotin-conjugated polyclonal rabbit antibody anti-PTX3 (500 ng/mL) followed by horseradish peroxidase-linked avidin (BIOSPA, Italy). Absorbance values were measured at *A*_450nm_ after the addition of tetramethylbenzidine substrate (TMB; Sigma-Aldrich/Merck). Murine PTX3 (DuoSet ELISA, #DY2166; R&D Systems), serum amyloid P component (SAP; ELISA; #MPTX20; R&D Systems), and myeloperoxidase (MPO; DuoSet ELISA, #DY3667; R&D Systems) levels were measured in mouse ACD-plasma after collection of blood from the cava vein.

### 2.10 Real-time PCR

Real-time PCR experiments were performed in extracted RNA from homogenized organs using the SybrGreen PCR Master Mix (Applied Biosystems, USA) in a 7900HT fast real-time PCRsystem (Applied Biosystems) in accordance with the manufacturer’s instructions (Applied Biosystems, USA) and the following primers: *Il-6*, Fw-5′-CCGGAGAGGAGACTTCACAG-3′, Rev-5′-CAGAATTGCCATTGCACAAC-3′; *Il-1β,* Fw-5′-GAGTGTGGATCCCAAGCAAT-3′, Rev-5′-TACCAGTTGGGGAACTCTGC-3′; *Tnf-α,* Fw-5′-CCACCACGCTCTTCTGTCTA-3′, Rev-5′-AGGGTCTGGGCCATAGAACT-3′; *Cox-2*, Fw-5′-CCCCCACAGTCAAAGACACT-3′, Rev-5′-GGCACCAGACCAAAGACTTC-3′; and *Gapdh*, Fw-5′-GGCATTGCTCTCAATGACAA-3′, Rev-5′-ATGTAGGCCATGAGGTCCAC-3′. Results are averaged absolute values of a triplicate for each organ analyzed with the Δ^2^CT method and normalized on GAPDH expression.

### 2.11 Statistics

Values are presented as mean ± SEM. The number of mice per experimental group is reported in figure legends. Student’s *t*-tests were performed after data were confirmed to fulfill the criteria of normal Gaussian distribution and equal variance (D’Agostino-Pearson omnibus normality test). Otherwise, Mann–Whitney test was applied. The ROUT method (*Q* = 1%) was used to identify outliers in the data groups. Analyses were performed with GraphPad Prism software (version 7.0c, GraphPad, USA). Differences were considered statistically significant when *p* < 0.05.

## 3 Results

### 3.1 Localization of PTX3 around initial lymphatic capillaries and sprouts

**Figure 1:**
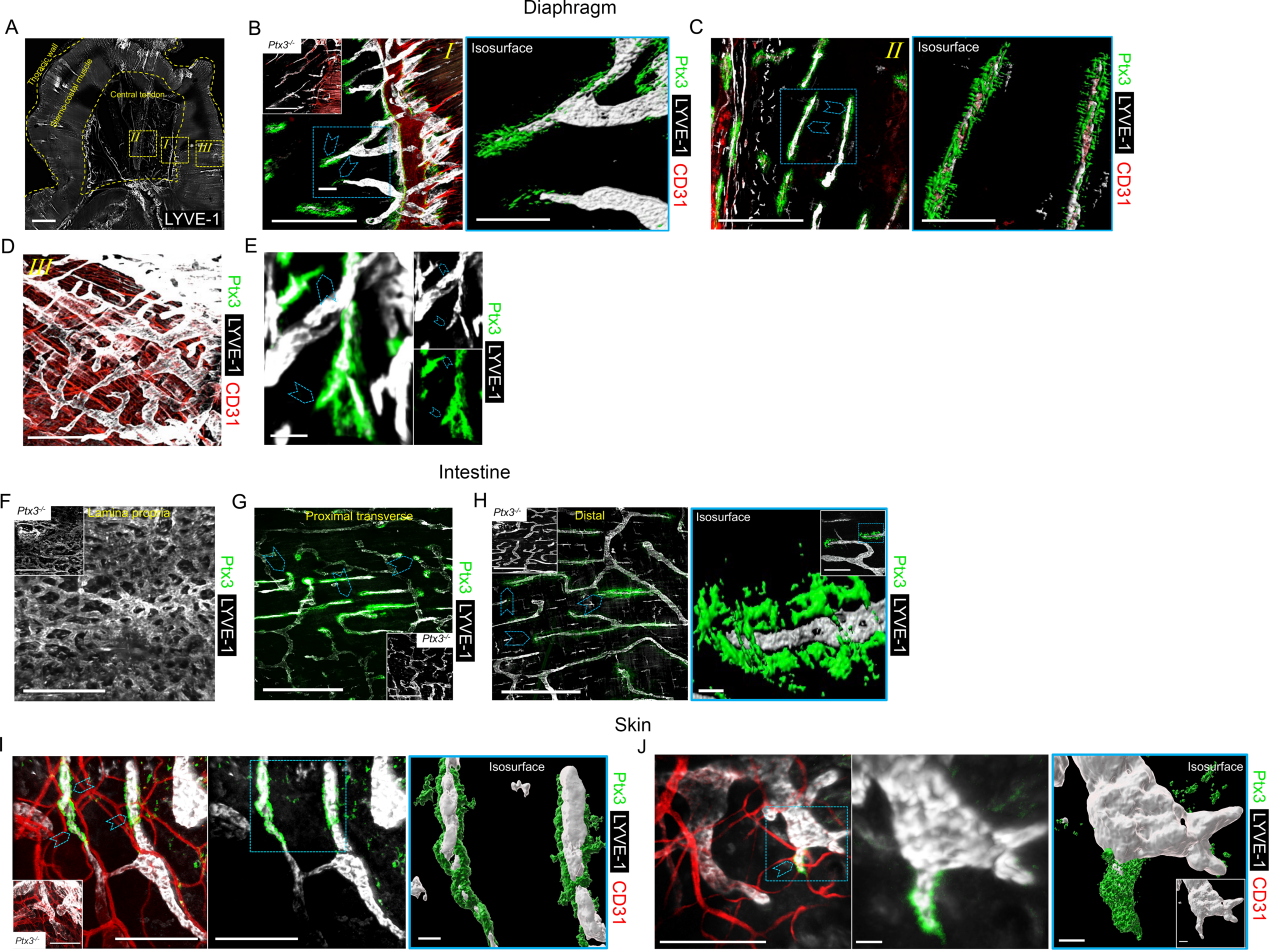
Localization of PTX3 in murine lymphatic vessels. **(A–H)** Whole mount immunofluorescence of the diaphragm, intestine, and ear skin analyzed in WT and *Ptx3^−/−^* mice. **(A)** Maximum intensity projection (MIP) of a mosaic image showing LYVE-1^+^ (white) LV distribution in whole mouse diaphragm. LVs extended radially and in parallel from thoracic wall to central tendon forming a reticular arrangement. Central tendon contained LYVE-1^+^ irregularly shaped lymphatic lacunae connected with mesothelium. Scale bar = 1 mm. **(B–D)** Close-up images of the yellow dashed areas *I*, *II*, and *III* of **(A)** analyzed after staining for LYVE-1 (white), CD31 (red), and PTX3 (green). **(B)** Close-up image of **(A)** showing the muscle–tendon interface (*I*); inset, *Ptx3^−/−^* diaphragm as control; **(C)** close-up images of **(A)** showing tendon (*II*); **(D)** close-up images of **(A)** showing peripheral muscle (*III*). *N* = 8 (WT) and 6 (*Ptx3^−/−^*) mice on 129/SV background; similar results were obtained in C57BL/6J mice (*n* = 6/genotype). (**B**, **C**, right) 3D isosurface rendering of confocal *z*-stacks of correspondent blue dashed area of **(B, C)**. Blue arrowheads indicate PTX3 around blind-ended LVs protruding toward central tendon **(B, *I*)** and around lacunae (**C**, *II*). B, C, left, D, scale bar = 500 µm; B, C, right, scale bar = 100 µm. **(E)** MIP image of representative of PTX3^+^ ECM around LYVE-1^+^ LEC sprouting in diaphragm. Scale bar = 10 µm. **(F–H)** MIP images of LYVE-1^+^ (white) LVs and PTX3 (green) localization in lymphatic plexus of distal colon *laminae* **(F)**, proximal transverse intestine submucosa **(G)**, and distal colon submucosa **(H)**. Insets in **F–H**, *Ptx3^−/−^* mice as control. *N* = 8 (WT) and 6 (*Ptx3^−/−^*) mice on 129/SV background; similar results were obtained in C57BL/6J mice (*n* = 6/genotype). Blue arrowheads indicate PTX3 associated with LYVE-1^+^ terminal LV capillaries in submucosa of proximal transverse **(G)** and distal colon **(H)**. (**H**, right) close-up MIP fluorescence image of blind-ended terminals in colon submucosa (inset) and 3D isosurface reconstruction of fluorescence signals in the blue dashed area. Scale bar = 500 µm (**F**, **G**, **H**, left), 10 µm (**H**, right), 100 µm (**H**, right, close-up). **(I, J)** Examples of MIP image of PTX3^+^ (green) ECM around LYVE-1^+^ (white) LVs **(I)** and LEC sprouting **(L)** in ear skin observed in the inner side. Blue arrowheads indicate PTX3 around blind-ended LVs **(I)** and cell sprouting **(J)**. (**I**, **J**, middle) Extracted signals of LYVE-1 and PTX3. (**I**, **J**, right) 3D isosurface rendering of *z*-stack images of correspondent blue dashed area of **I** (left) and **J (**left) (*n* = 4 WT, 3–5 *z*-stack confocal images for each). (**I**, inset) *Ptx3^−/−^* skin (*n* = 3) as control. Scale bar = 100 µm (**I**, **J**, left and **I** middle) and 10 µm (**J**, middle and **I**, **J**, right).

Previous studies had shown that PTX3 transcripts are constitutively expressed by human and mouse *in vitro* cultured LEC and in mouse LEC clusters with specialized functions (8, 12, 55–58). We therefore set out to investigate the localization and functional significance of PTX3 in LVs, taking advantage of gene-modified mice. Protocols were then designed to simultaneously evaluate the stereological localization of PTX3 in a 3D visualization of the complete lymphatic and blood vasculature in whole mounted mouse organs (**Supplementary Figure S1**, **Supplementary Videos S1**, **S2**). Diaphragm, trachea, intestine, and ear skin were used for this purpose because they have been extensively utilized to investigate the molecular organization of LVs (61, 65, 66). In the diaphragm (**Figure 1A**), PTX3 selectively localized around LYVE-1^+^ initial LVs protruding at the interface between the peripheral muscle and the central tendon and towards the peritoneal cavity through lymphatic stomata located between the mesothelial cell layer (62) (**Figure 1B**, **Supplementary Video S3**). Moreover, PTX3 circumferentially encapsulated LYVE-1^+^ lymphatic lacunae (62) in the central tendon (**Figure 1C**). As assessed by 3D isosurface rendering analysis (**Figures 1B, C**, **Supplementary Video S3**), PTX3 was enriched around the terminal part of blind-ended LV capillaries at the LEC-ECM interface. CD31^+^ blood vessels were negative (**Figures 1B, C**). In contrast, PTX3 was not associated with LVs in the muscular portion that extend radially and parallel from the thoracic wall to the central tendon (62) (**Figure 1D**). When evident under normal conditions (67), single LEC sprouts were surrounded by PTX3-rich ECM (**Figure 1E**, **Supplementary Video S4**). In the diaphragm, PTX3 expression surrounded the tips of the terminal LVs and *lacunae*, where F4/80^+^ tissue-resident macrophages were present (**Supplementary Figure S2**, **Supplementary Video S5**). In the proximal transverse and distal intestine, PTX3 expression was restricted to LYVE-1^+^ LVs of the submucosa, whereas the plexus of the lamina propria was negative (**Figures 1F–H**). PTX3 was associated with 20%–30% of terminal LV (**Figures 1G, H**), localized around blind-end tips associated with the ECM (**Figure 1H**). Similarly, in the skin, PTX3 localized circumferentially in the terminal LVs at the interface with LECs and the surrounding ECM (**Figure 1I**), as well as in the sprouting tips of LECs (**Figure 1J**). In the trachea, PTX3 was localized around blind tips of LYVE-1^+^ LVs in the mucosa between cartilaginous rings (**Supplementary Figure S3**). Therefore, PTX3 was constitutively expressed in LVs with a selective and conserved localization in different organs in blunt-ended terminals of LV capillaries at the interface between LEC and ECM.

**Figure 2:**
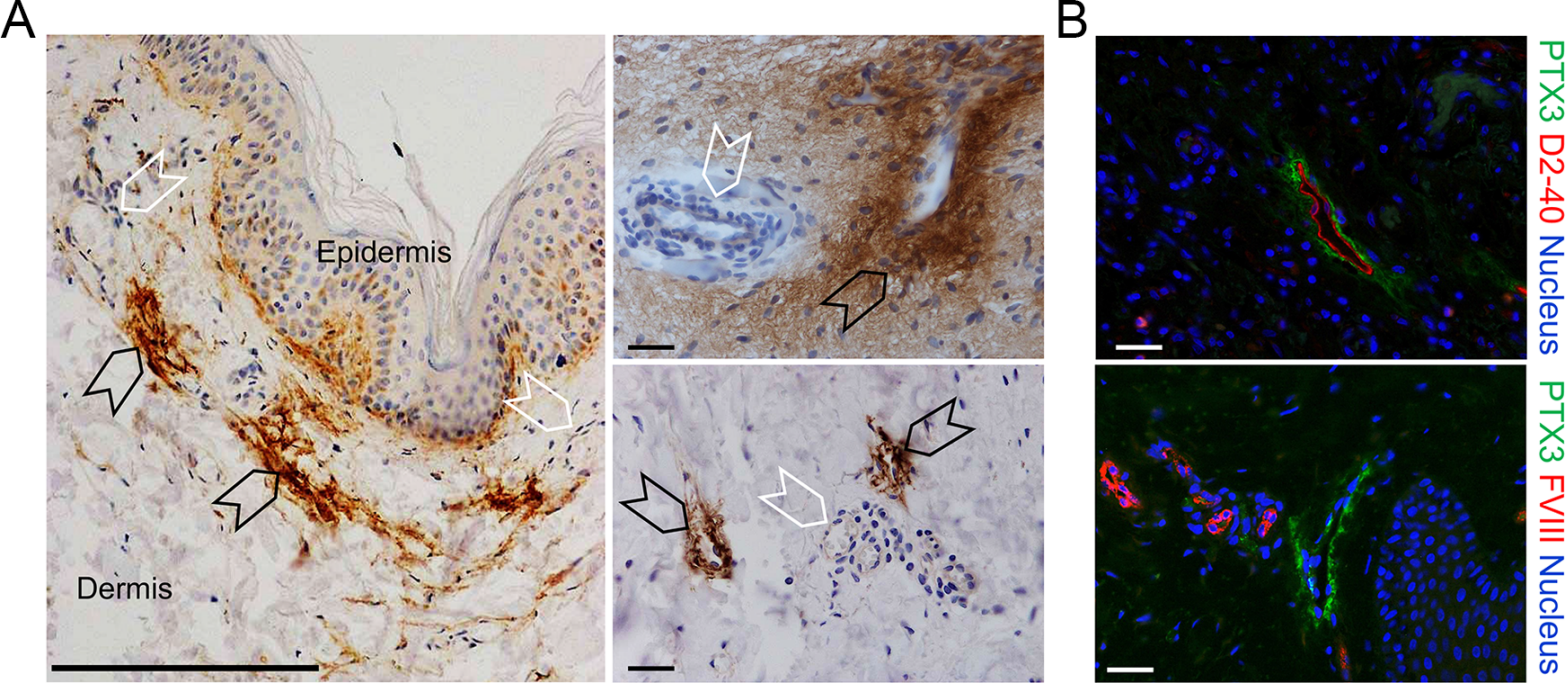
Localization of PTX3 in human lymphatic vessels. **(A, B)** Classic immunohistochemistry and multifluorescence microscopy analysis of normal human skin samples. **(A)** Images are representatives of *n* = 3 independent donors. Left, PTX3 (DAB, brown) localization at ECM around LVs (black arrowheads) of the dermis counterstained with H&E. Blood vessels (white arrowheads) resulted negative to PTX3 staining. Right, acquisitions at different magnification of LVs (black arrowheads) and blood vessels (white arrowheads). Scale bar = 100 µm. **(B)** Immunofluorescence analysis on normal skin confirming PTX3 positivity in the perilymphatic interstitium while blood vessels are negative. PTX3 staining is in green, LECs are identified by positivity with human D2-40 (upper right panel), and vascular ECs are recognized by positivity to Factor VIII (lower right panel), both in red. DAPI is used for nuclear staining (blue). Scale bar = 10 µm.

It was important to assess whether PTX3 is expressed in human LVs. As shown in **Figure 2**, as assessed by IHC, PTX3 was found to surround LVs in human skin, similarly to what was observed in the mouse. A similar pattern was observed in the stomach, large and small intestines, lung, prostate, and breast (not shown).

### 3.2 PTX3 is essential for shaping the lymphatic vasculature

**Figure 3:**
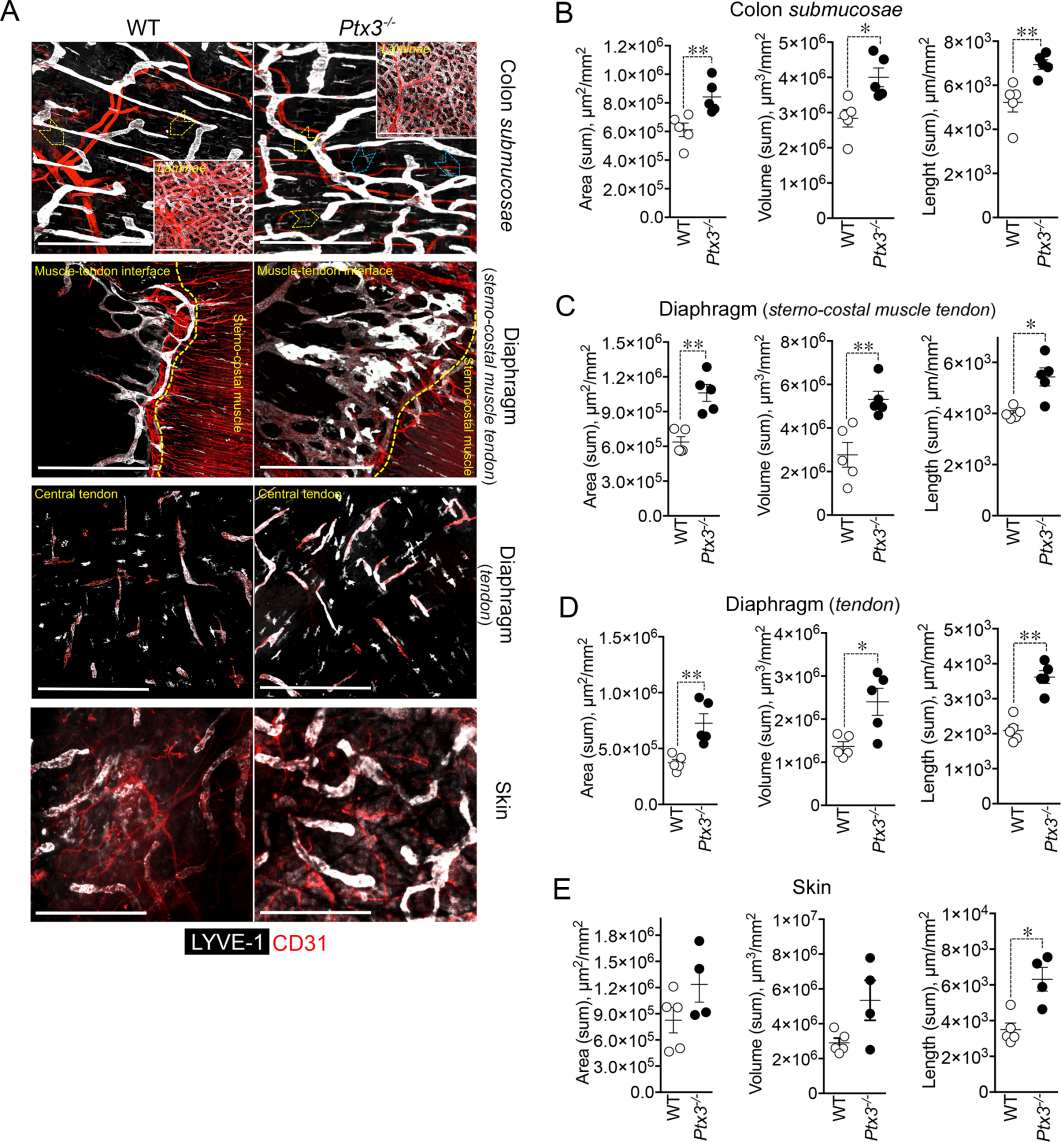
PTX3 deficiency is associated with morphometric alterations of terminal lymphatic capillaries. **(A)** Upper panel, MIP of confocal *z*-stack images showing CD31^+^ (red) blood and LYVE-1^+^ (white) lymphatic vasculature in colon submucosa and lamina (inset) of WT (*n* = 5) and *Ptx3^−/−^* (*n* = 5) mice on 129/SV background. Yellow arrowheads indicate blind-ended terminal LV capillaries; blue arrowheads, disorganized LV pattern and anastomosis in *Ptx3^−/−^* mice. Middle panels, MIP of confocal *z*-stack images of diaphragm at the muscle–tendon interface (middle; *n* = 5 WT and *Ptx3^−/−^* mice) and tendon (lower; *n* = 5 WT and *Ptx3^−/−^* mice). Yellow dashed line traces the demarcation between sterno-costal muscle and tendon. Lower panel, MIP of confocal *z*-stack images showing LVs of the ear skin (*n* = 5 WT; *n* = 4 *Ptx3^−/−^* mice). Scale bar = 800 µm. **(B–E)** Quantification of **(A)** as expression of area (µm^2^/mm^2^), volume (µm^3^/mm^2^), and length (µm/mm^2^) of LYVE-1^+^ LVs in colon submucosa **(B)**, diaphragm at the muscle–tendon interface **(C)**, diaphragm tendon **(D)**, and skin **(E)**. Each spot refers to a mean of *n* = 3–7 **(B)**, *n* = 2–6 **(C)**, *n* = 3–5 **(D)**, or *n* = 1–6 **(E)** 3D reconstructed *z*-stack images of 1.65 mm^2^ of each single mouse and expression of mean±SE per mm^2^. *p < 0.05; **p < 0.01 (two-tailed, Mann–Whitney test).

Having found that PTX3 is localized in the ECM of LV, it was important to investigate its function in the structural organization of the lymphatic vasculature, taking advantage of PTX3 gene-targeted mice (40). Morphometric analysis was conducted in whole mounted colon and diaphragm of WT and *Ptx3*-deficient adult mice (**Figure 3**). In the colon submucosa, the *Ptx3*-deficient lymphatic vasculature appeared disorganized with the increased frequency of abnormal anastomosis and the number of blind-ended capillaries compared to WT [*n* = 9.20 ± 1.37 vs. 6.32 ± 0.47/mm^2^ (mean ± SE), *Ptx3^−/−^* vs. WT (*n* = 5); *p* = 0.04 (two-tailed, Mann–Whitney test); not shown]. Moreover, LVs were enlarged, and terminal capillary end tips were more rounded and apparently less projected toward the ECM (**Figure 3A**). In *Ptx3*-deficient mice, a general augmentation in density of LVs was measured as LYVE-1^+^ area, volume, and length (**Figure 3B**, **Table 1**). No difference was instead observed in the LV plexus of the colon lamina (**Figure 3A**, inset). Similar abnormalities were observed in the *Ptx3*-deficient diaphragm, consistently with augmentation and disorganization of protruding LVs at the interface between peripheral sterno-muscle and tendon (**Figure 3A**). At the muscle–tendon interface, the area, volume, and length of the LYVE-1^+^ LV area were significantly increased (**Figure 3C**, **Table 1**). In the tendon, an increased number and dimension of LYVE-1^+^ *lacunae* was also observed in *Ptx3*-deficient mice (**Figures 3A, D**, **Table 1**). Similarly, the density of LVs in *Ptx3^−/−^* mice was increased in the skin (**Figures 3A, E**, **Table 1**). Thus, alterations observed in the terminal LV tips and hyperplasia of the lymphatic vascular network associated with PTX3 deficiency were consistent across all examined organs.

**Table 1:** Morphometric analysis of LVs in *Ptx3*-deficient mice^1^.

Organ	Genotype	Area (µm^2^/mm^2^)	Volume (µm^3^/mm^2^)	Length (µm/mm^2^)
Colon submucosa	WT	6.12 ± 0.47 × 10^5^	2.84 ± 0.25 × 10^6^	5.22 ± 0.43 × 10^3^
	*Ptx3^−/−^*	8.41 ± 0.51 × 10^5***^	4.0 ± 0.26 × 10^6*^	6.94 ± 0.22 × 10^3***^
	*Prox-1-^Cre-^/Ptx3^flox/flox^*	0.92 ± 0.04 × 10^6^	2.06 ± 0.15 × 10^6^	4.64 ± 0.19 × 10^3^
	*Prox-1-^Cre+^/Ptx3^flox/flox^*	1.25 ± 0.08 × 10^6§^	2.68 ± 0.15 × 10^6^	6.47 ± 0.48 × 10^3§§^
Diaphragm Muscle–tendon interface	WT	0.64 ± 0.05 × 10^6^	2.77 ± 0.57 × 10^6^	4.01 ± 0.01 × 10^3^
	*Ptx3^−/−^*	1.06 ± 0.07 × 10^6***^	5.31 ± 0.37 × 10^6***^	5.43 ± 0.35 × 10^3*^
	*Prox-1-^Cre-^/Ptx3^flox/flox^*	4.04 ± 0.25 × 10^5^	2.26 ± 0.40 × 10^6^	2.07 ± 0.29 × 10^3^
	*Prox-1-^Cre+^/Ptx3^flox/flox^*	5.20 ± 0.23 × 10^5§^	3.28 ± 0.14 × 10^6§^	2.78 ± 0.34 × 10^3^
Diaphragm Tendon	WT	3.72 ± 0.31 × 10^5^	1.37 ± 0.11 × 10^6^	2.09 ± 0.15 × 10^3^
	*Ptx3^−/−^*	7.27 ± 0.85 × 10^5***^	2.40 ± 0.31 × 10^6**^	3.62 ± 0.18 × 10^3***^
Skin	WT	8.26 ± 1.46 × 10^5^	2.91 ± 0.27 × 10^6^	3.50 ± 0.37 × 10^3^
	*Ptx3^−/−^*	12.38 ± 2.05 × 10^5^	5.34 ± 1.68 × 10^6^	6.32 ± 0.67 × 10^3**^

^1^Data are presented as mean ± SEM. *Ptx3^−/−^* vs. WT: ^*^ *p* = 0.02; ^**^ *p* = 0.03; ^***^ *p* = 0.008. *Prox-1-^Cre+^/Ptx3^flox/flox^* vs. *Prox-1-^Cre-^/Ptx3^flox/flox^*: *^§^p* = 0.03; ^§§^ *p* = 0.057 (two-tailed Mann–Whitney test).

**Figure 4:**
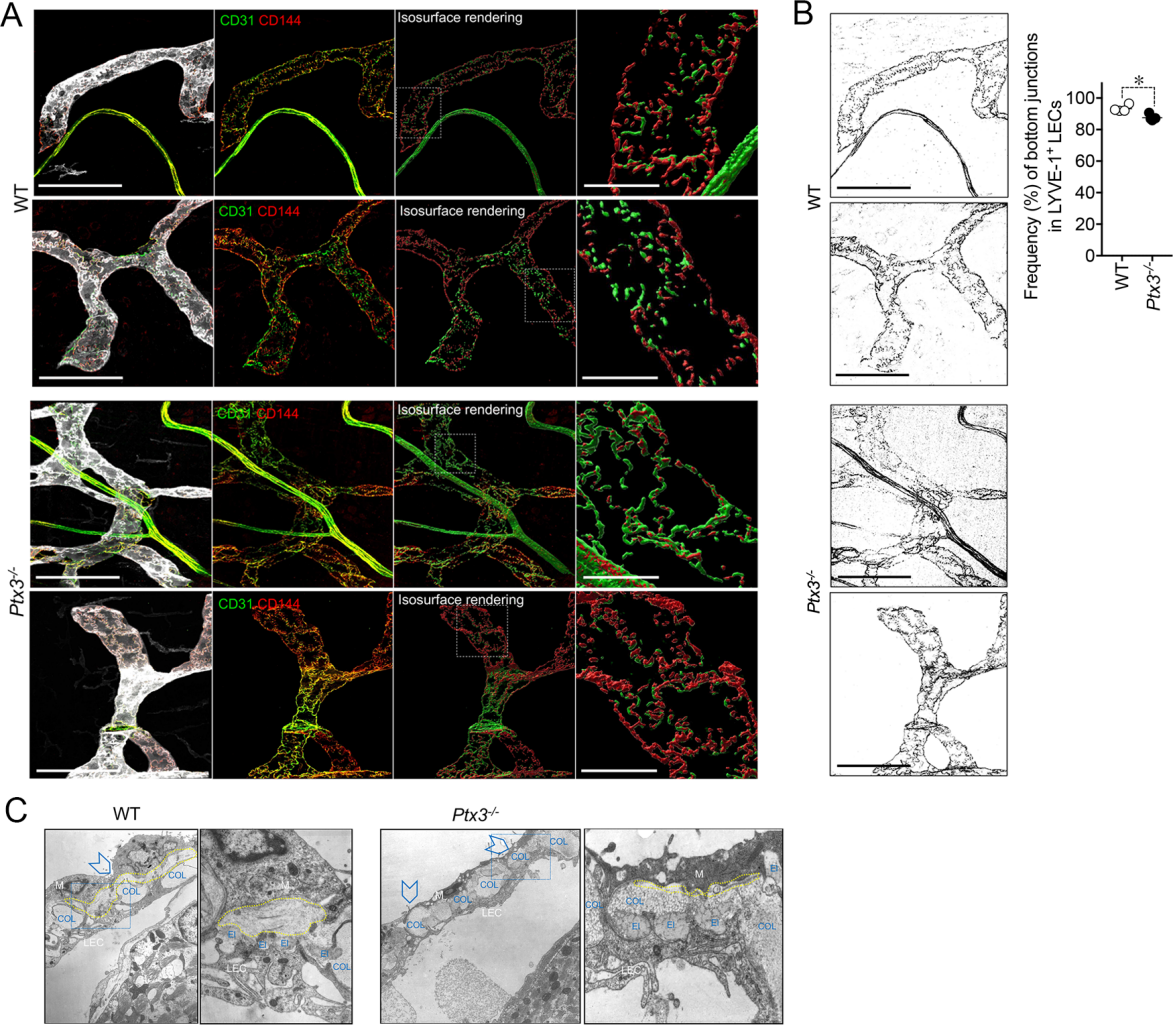
PTX3 deficiency is associated with alterations in LEC junctions at initial lymphatics. **(A)** Representative MIP images of *z*-stack images referring two mice/genotype of the distribution of CD31 (green) and CD144 (red) on LYVE-1^+^ (white) terminal LVs in colon submucosa are shown. From left to right for each mouse, the following are shown: MIP images of the contribution of fluorescence signals; extracted fluorescence signal related to LEC junctions (CD31 and CD144); 3D isosurface rendering aimed at reconstituting LEC junction distribution; relative close-up of the area delimited by white dotted line showing junction distribution on single LECs. Scale bar = 100 µm (left) and 10 µm (right). **(B)** CD144^+^ signal extracted from the corresponding MIP images of **(A)** and used for the quantification of zipper vs. button junctions (left). Quantification of zipper vs. button junctions in WT (*n* = 4) and *Ptx3^−/−^* (*n* = 5) mice on 129/SV background (right). Each spot refers to the mean ± SE of two to three images of 0.32 mm^2^ per mouse colon and results are expressed as % of LECs with button (discontinuous staining of CD144 for each LEC) junctions on total LYVE-1^+^ LECs (87.48 ± 0.83% vs. 93.25 ± 1.09%, *Ptx3^−/−^* vs. WT; *p < 0.05; two-tailed, Mann–Whitney test). **(C)** Structural alterations in the perilymphatic ECM of *Ptx3*-deficient mice. EM micrographs of terminal LVs forming *stomata* in the diaphragm of WT (left; *n* = 3) and *Ptx3^−/−^* (right; *n* = 3) mice. In WT mice, organization of the different ECM components around stomata (arrowheads) and in peri-stomata areas appears as an ordinate alternation of elastic (El) and collagen (COL) fibers, and pericellular spaces (outlined in yellow) containing non-fibrillar ECM. *Ptx3*-deficient mice showed a reduction in HA-rich pericellular ECM (outlined in yellow). Left panels, ×9,000 magnification; right panels, ×36,000 magnification of the blue dashed areas. El, elastic fibers; COL, collagen fibers; LEC, lymphatic EC; M, mesothelial cells.

Differences in the distribution of tight junction-associated molecules and VE-cadherin define specialized functions of LVs (33). In adults, initial LVs show button junctions as opposed to the zippers of collecting vessels (32, 61), both subject to transient changes (buttons-to-zippers) in inflammation (61). The ECM regulates the state of LEC junctions through integrin-mediated signals (33, 37). As ascertained by the analysis of VE-cadherin (CD144) distribution on LECs (**Figures 4A, B**), *Ptx3*-deficient mice showed decreased button junctions at initial lymphatics compared to WT in the colon submucosa. The alterations associated with PTX3 deficiency in LVs were not dependent on a different basal state of inflammation in the tissue, as ascertained by measuring the local expression of key cytokines in modulating the morphometric appearance and function of LVs (e.g., IL-1β and TNF-α) (68) (**Supplementary Figure S4A**). In agreement, a similar abundance of tissue-resident immune cells in proximity of LVs in the basal state was observed in *Ptx3*-deficient mice compared to WT (**Supplementary Figure S4B**). Moreover, similar blood levels of SAP, a major acute-phase protein in mice, and MPO, an expression of neutrophil basal activation, were observed (**Supplementary Figure S4C**).

Ultrastructural studies of perilymphatic ECM around terminals in the diaphragm revealed differences in the organization of lymphatic *stomata* and peri-*stomata* areas in *Ptx3*-deficient mice consisting in the disorganized alternation of elastic and collagen fibers and the reduction of HA in the non-fibrillar ECM of pericellular spaces (**Figure 4C**), thus supporting PTX3 as a constituent of viscoelastic ECM capable of organizing and remodeling the tissue beyond its function in innate immunity.

### 3.3 Cellular source of LV-organizing PTX3

**Figure 5:**
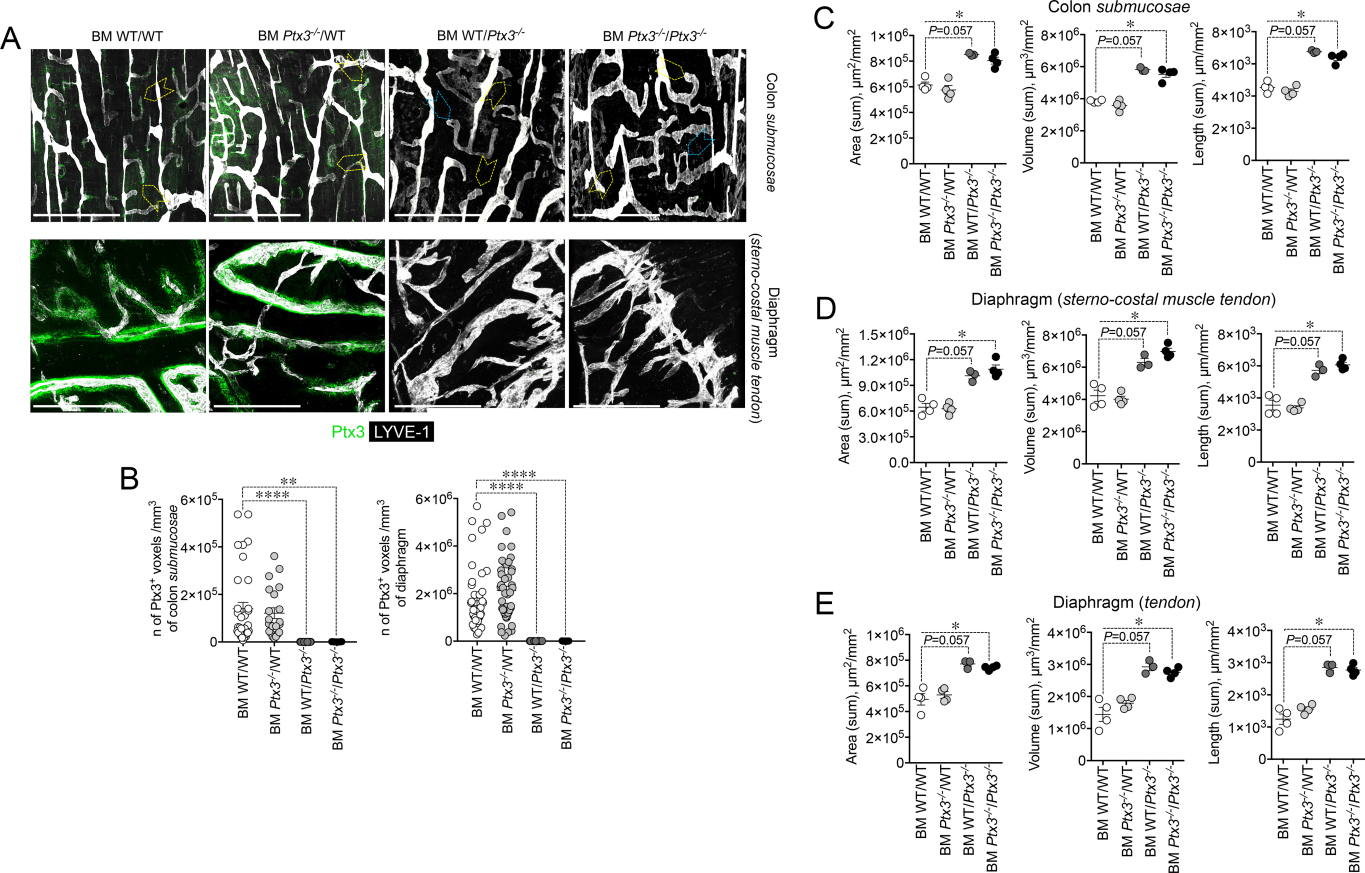
Deficiency in stromal PTX3 is responsible of the morphometric alterations of terminal lymphatic capillaries. **(A)** MIP of *z*-stack images showing expression and localization of PTX3 (green) in LYVE-1^+^ (white) LVs of WT and *Ptx3^−/−^* mice receiving WT and *Ptx3^−/−^* BM, respectively (*n* = 3–4 mice/group; C57BL/6J). Upper panel, colon submucosa. Yellow arrowheads indicate blind-ended terminal LV capillaries; blue arrowheads, alterations in blind ends of LVs in mice deficient in PTX3 in the stroma. Lower panel, *z*-stack images of the diaphragm at the muscle–tendon interface of different chimeric groups. Scale bar = 800 µm. **(B)** Quantification of PTX3^+^ voxels around LYVE-1^+^ LVs after 3D rendering. Results are expressed as mean ± SE of PTX3^+^ voxels/mm^3^. Each spot corresponds to quantification of a single MIP image of 1.20 mm^2^ [(colon: *n* = 34 BM WT/WT*, n* = 21 BM *Ptx3^−/−^*/WT, *n* = 23 BM WT/*Ptx3^−/−^*, *n* = 15 BM *Ptx3^−/−^*/*Ptx3^−/−^*; diaphragm: *n* = 45 BM WT/WT, *n* = 46 BM *Ptx3^−/−^*/WT, *n* = 24 BM WT/*Ptx3^−/−^*, *n* = 29 BM *Ptx3^−/−^*/*Ptx3^−/−^*) *n* of images] of *n* = 3 (BM WT/*Ptx3^−/−^*) and 4 (BM WT/WT; BM *Ptx3^−/−^*/WT; BM *Ptx3^−/−^*/*Ptx3^−/−^*) mice/group. **p < 0.01; ****p < 0.0005 (two-tailed; unpaired *t*-test). **(C–E)** Morphometric analysis of **(A)** after 3D isosurface rendering of *z*-stack images expressed as area (µm^2^/mm^2^), volume (µm^3^/mm^2^), and length (µm/mm^2^) of LYVE-1^+^ LVs in colon submucosa **(C)**, diaphragm at the muscle–tendon interface **(D)**, and diaphragm tendon **(E)**. Each spot refers to the mean of *n* = 4–25 **(C)**, *n* = 2–9 **(D)**, or *n* = 3–12 **(E)** 3D reconstructed *z*-stack images of 1.2 mm^2^ of each single mouse and expressed as mean ± SE per mm^2^. *p < 0.05 (two-tailed, Mann–Whitney test).

PTX3 is produced by a variety of cell types including ECs and BM-derived myeloid cells (48). In an effort to define the cellular source of the LV-organizing PTX3, we took advantage of BM chimeric mice and of *Cre* recombinase-expressing inducible mice with selective *Ptx3* deletion in LECs (*Prox-1-^Cre^/Ptx3^flox/flox^*) (60). In the BM transplantation setting, PTX3 expression around initial lymphatic capillaries was abolished in mice deficient in stromal PTX3 (**Figures 5A, B**). Morphometric defects observed in *Ptx3*-deficient mice were recapitulated in the recipient mice lacking PTX3 in the stroma, both in the colon submucosa (**Figures 5A, C**) and in the diaphragm (**Figures 5A, D, E**).

**Figure 6:**
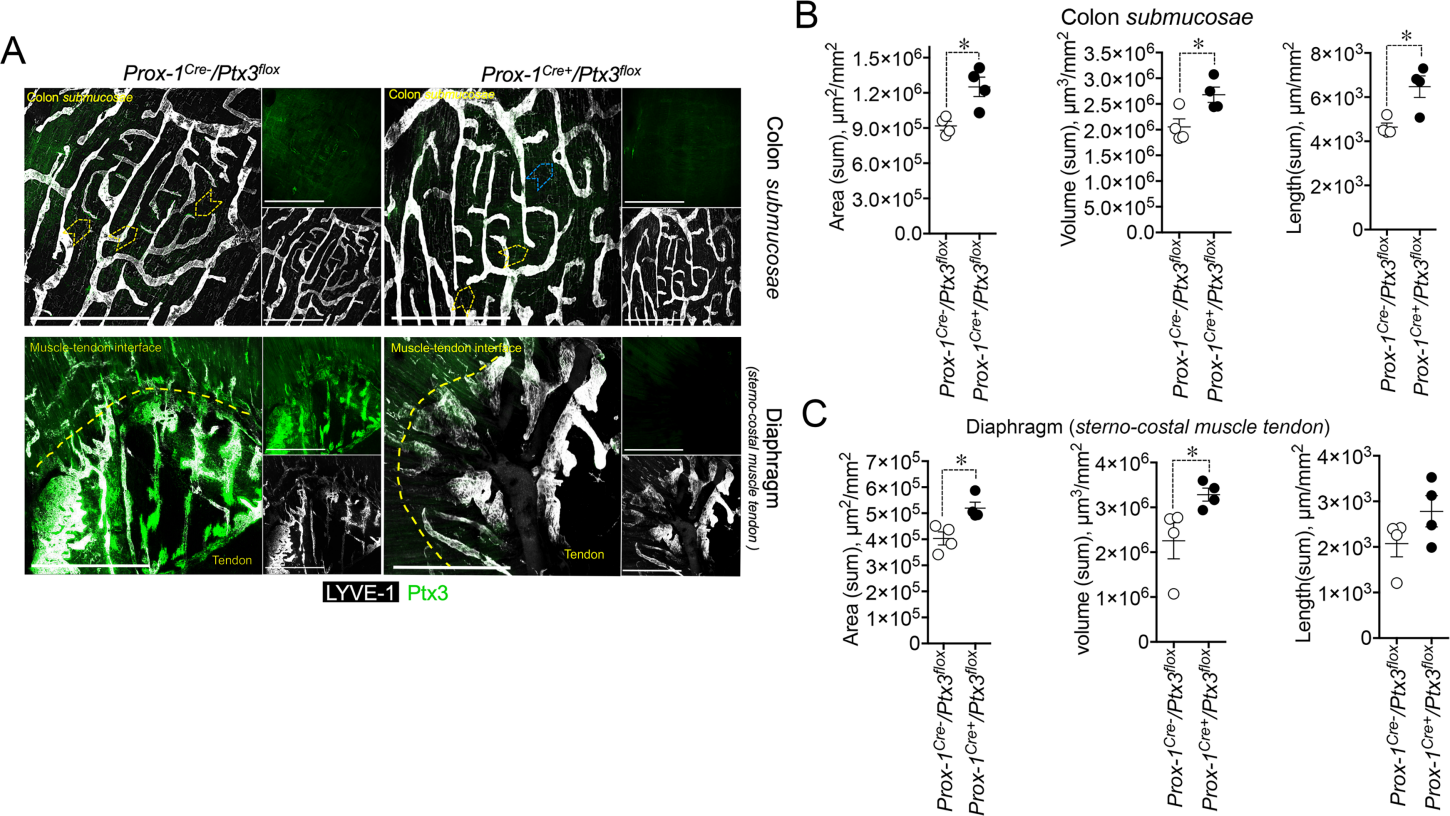
LEC-restricted PTX3 deficiency recapitulates the morphometric alterations at terminal lymphatic capillaries. **(A)** MIP of *z*-stack images showing the expression and localization of PTX3 (green) in LYVE-1^+^ (white) LVs of *Prox-1-^Cre-^/Ptx3^flox/flox^* and *Prox-1-^Cre+^/Ptx3^flox/flox^* mice (*n* = 4/genotype; C57BL/6J). Upper panel, colon submucosa; lower panel, diaphragm at the tendon–muscle interface. Yellow arrowheads indicate blind-ended terminal LV capillaries; blue arrowheads, morphometric alterations in blind ends of LVs of *Prox-1-^Cre+^/Ptx3^flox/flox^* mice. Scale bar = 800 µm. **(B, C)** Morphometric analyses after 3D rendering of *z*-stack images of area (µm^2^/mm^2^), volume (µm^3^/mm^2^), and length (µm/mm^2^) of LYVE-1^+^ LVs in colon submucosa **(B)** and diaphragm at the muscle–tendon interface **(C)**. Each spot refers to the mean ± SE of 2–10 (colon submucosa) or 1–12 (diaphragm) MIP images of 1.20 mm^2^ per mouse tissue and presented per mm^2^. *p < 0.05 (two-tailed, Mann–Whitney test).

As shown in **Figure 6A**, PTX3 deficiency in LVs (*Prox-1-^Cre+^/Ptx3^flox/flox^*; *n* = 4) resulted in decreased expression of the protein in closed terminal lymphatic capillaries, both in the colon submucosa and the diaphragm compared with the genetic controls (*Prox-1-^Cre-^/Ptx3^flox/flox^*; *n* = 4). Morphological and organization abnormalities in LVs were also observed in *Ptx3* conditional mice (**Figures 6A–C**) though less prominent than in *Ptx3^−/−^* animals. In the colon submucosa, the LV density in *Prox-1-^Cre+^/Ptx3^flox/flox^* mice was increased compared with controls, as ascertained by measuring the area, volume, and length (**Figure 6B**, **Table 1**). The number of blind-ended lymphatic capillaries was not different between genotypes [*n* = 6.50 ± 0.29 vs. 5.25 ± 0.25/mm^2^ (mean±SE); not shown], although in *Prox-1-^Cre+^/Ptx3^flox/flox^* mice, lymphatic capillaries retained an apparent morphology that suggest lower ECM penetrating capacity showing more rounded blind ends compared to *Prox-1-^Cre-^/Ptx3^flox/flox^* mice (**Figure 6A**). In the same line, LVs at the interface between the diaphragm peripheral sterno-muscle and tendon appeared dilated and disorganized in *Prox-1-^Cre+^/Ptx3^flox/flox^* mice compared with controls (**Figure 6A**) and of increased density, as assessed by measurement of the area, volume, and length (**Figure 6C**, **Table 1**). Thus, LECs are a major source of PTX3 localized in the surrounding ECM, and LEC-derived PTX3 plays a key role in shaping LVs.

### 3.4 Impact on LV functions

**Figure 7:**
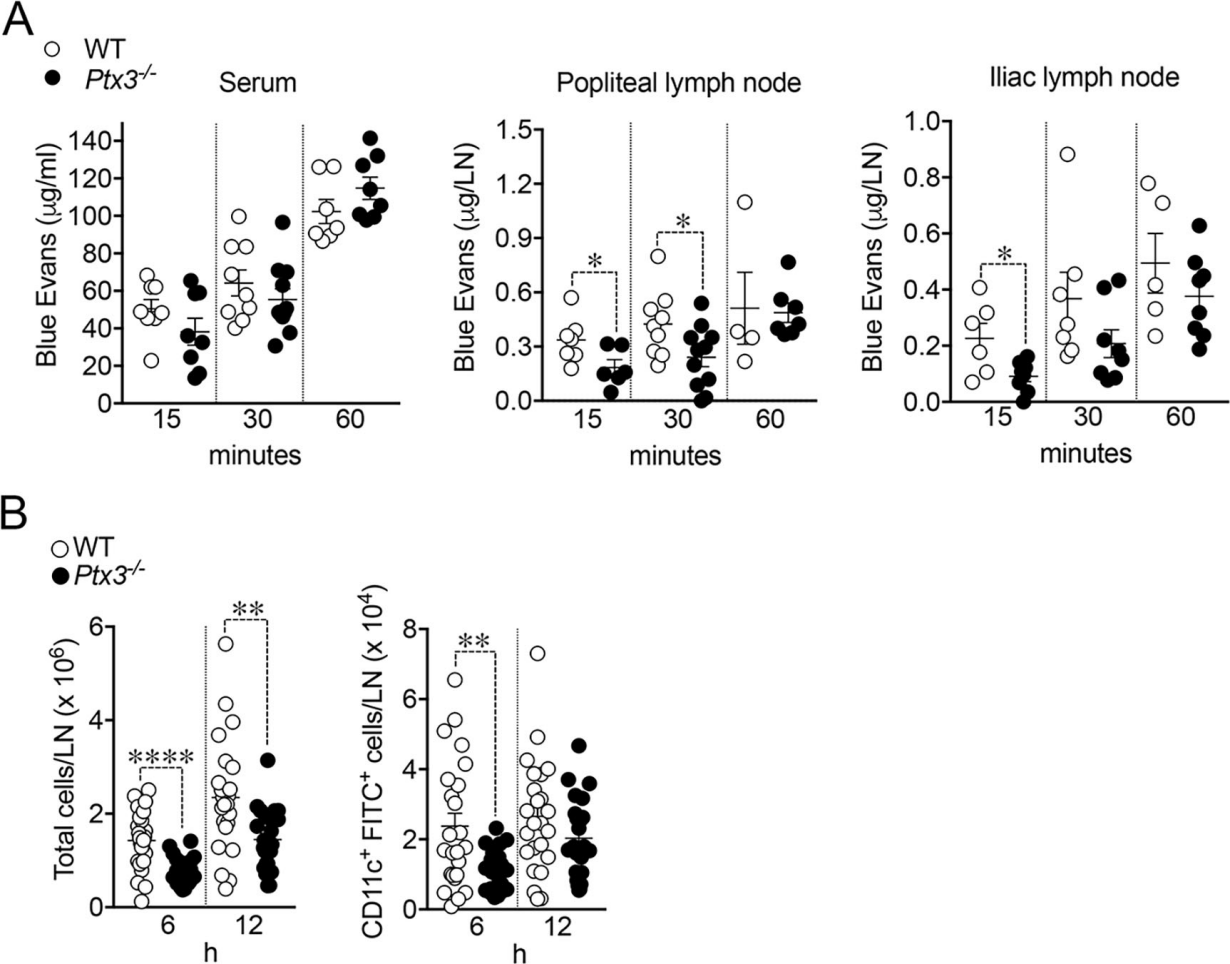
Impaired fluid drainage and DC trafficking. **(A)** Lymphatic fluid drainage. Evans Blue (EB) dye was injected into the foot pads of WT and *Ptx3*-deficient animals (129/SV) and drained EB was quantified at different time points in serum (left), popliteal (middle), and iliac (right) LNs. Each spot corresponds to a single animal and refers to the mean EB in the blood (38.22 ± 7.02 vs. 50.42 ± 5.02 µg/mL, *Ptx3^−/−^* vs. WT), or draining LN (at 15 min, 0.18 ± 0.04 vs. 0.34 ± 0.05 µg/pLN; 0.09 ± 0.02 vs. 0.23 ± 0.05 µg/iLN; at 30 min, 0.24 ± 0.05 vs. 0.42 ± 0.06 µg/pLN). Results refer to one independent experiment (15 and 60 min) or two pooled experiments (30 min) out of two (15 min) or three (30 min) performed. *p < 0.05 (two-tailed; unpaired *t*-test). **(B)** DC trafficking. Mice were painted with 0.2 mL of a 5 mg/mL FITC solution and inguinal LNs collected and disaggregated at indicated time points. LN cells were counted and analyzed by FACS. Results are reported as the number of CD11c and FITC double-positive DCs per LN (6 h, 1.13 ± 0.12 × 10^4^ vs. 2.37±0.37 × 10^4^ CD11c^+^FITC^+^ DCs/LN, *Ptx3^−/−^* vs. WT; 12 h, 2.03 ± 0.24 × 10^4^ vs. 2.62 ± 0.33 × 10^4^ CD11c^+^FITC^+^ DCs/LN, *Ptx3^−/−^* LNs). Each spot corresponds to a single LN. *N* = 12 mice/group at 6 and 12 h Two experiments pooled per time point out of four performed on C57BL/6J genetic background or four (6 h) and three (12 h) performed in 129/SV mice with similar results. **p < 0.01; ****p < 0.0005 (two-tailed; unpaired *t*-test).

In an effort to investigate the functional implications of the abnormal structure of *Ptx3*-deficient lymphatics, we measured fluid drainage and DC trafficking to LNs. The draining capacity was evaluated, measuring EB dye after injection into the foot pads of WT and *Ptx3*-deficient animals (**Figure 7A**). In resting conditions, *Ptx3^−/−^* mice did not display any apparent dysfunction in fluid drainage (e.g., spontaneous edema) as assessed by gross inspection and histology (not shown). As ascertained by the quantification of EB in serum, popliteal LN (pLN), and iliac LN (iLN) at different time points, *Ptx3^−/−^* mice showed defects in draining and transport of EB at 15 min in serum, pLN, and iLN, and at 30 min in pLN (**Figure 7A**). DC migration to the LN through LVs of WT and *Ptx3*-deficient mice was evaluated in a skin FITC painting model followed by FACS analysis of inguinal LN. As shown in **Figure 7B**, in *Ptx3^−/−^* mice, a decreased number of CD11c^+^FITC^+^ DCs was measured in inguinal LN in comparison with WT, while the frequency was similar (**Supplementary Figure S5**). In experiments not involving the use of FITC, the basal number of total cells was similar in the inguinal LN of different genotypes (WT, 2.01 ± 0.8 × 10^6^/LN, *n* = 7; *Ptx3^−/−^*, 1.47±0.6 × 10^6^/LN, *n* = 5) and CD11c^+^ DCs (WT, 5.02 ± 2.3 × 10^4^/LN, *n* = 7; *Ptx3^−/−^*, 3.78 ± 2.5 × 10^4^/LN, *n* = 5). Thus, the altered morphology and organization observed in the LVs of *Ptx3*-deficient hosts are reflected by alterations in fluid drainage and DC trafficking. Given the role of PTX3 in humoral innate immunity against diverse microbial agents (40, 69, 70), we tested its role in resistance to infection with *S. enterica* serovar Typhimurium, which spreads from the gastrointestinal tract via the lymphatic route (71). Using the ΔaroA auxotrophic *Salmonella* mutant strain, characterized by an attenuated ability to replicate *in vivo* (64), and measuring dissemination after oral infection, we found a higher number of bacteria in mesenteric LNs of *Ptx3*-deficient animals (**Supplementary Figure S6**). Given that PTX3 interacts with specific bacteria, including *Salmonella*, and plays a role in local innate immunity (41), further experiments are needed to investigate the relevance of the lymphatic system phenotypes associated with PTX3 deficiency.

## 4 Discussion

Several lines of evidence pointed to a role of PTX3 in LVs, including a constitutive expression in murine and human *in vitro* cultured LECs (55–57). More recently, single-cell RNA sequencing confirmed PTX3 constitutive expression in murine and human LECs (4, 7, 8, 58, 59). The power of computational methods has made it possible to identify distinct LEC subsets in both humans and mice characterized by distinct gene expression profiles and performing different biological functions (7, 8). Five different key subsets were identified among murine LECs. These include ceiling LECs (cLECs), floor LECs (fLECs), Ptx3-LECs, valve-LECs, and Marco-LECs. Each subset performs specific roles, with cLECs forming the ceiling of lymphatic sinuses, and fLECs contributing to the floor structure. Ptx3-LECs are characterized by *Ptx3* expression and are associated with capillary networks, while valve-LECs are involved in the formation of LV valves. Marco-LECs play a crucial role in immune regulation, particularly within the medullary sinus (8). In an oncogenic *PIK3CA*-dependent mouse model of LV malformations, *Ptx3* expression was associated with abnormal dermal LEC sprouts with a molecular distinction that includes enrichment for genes with high responsivity to VEGF-driving lymphangiogenesis and expression of an immunoregulatory profile (e.g., *Mrc1, Ccl21, Lyve1*) (58). Despite some differences in the complexity and specific gene expression profiles between human and murine LECs, these subsets are mostly conserved across species, and this suggests that a significant portion of morphological and functional data obtained from mouse models can be extended to humans.

**Figure 8:**
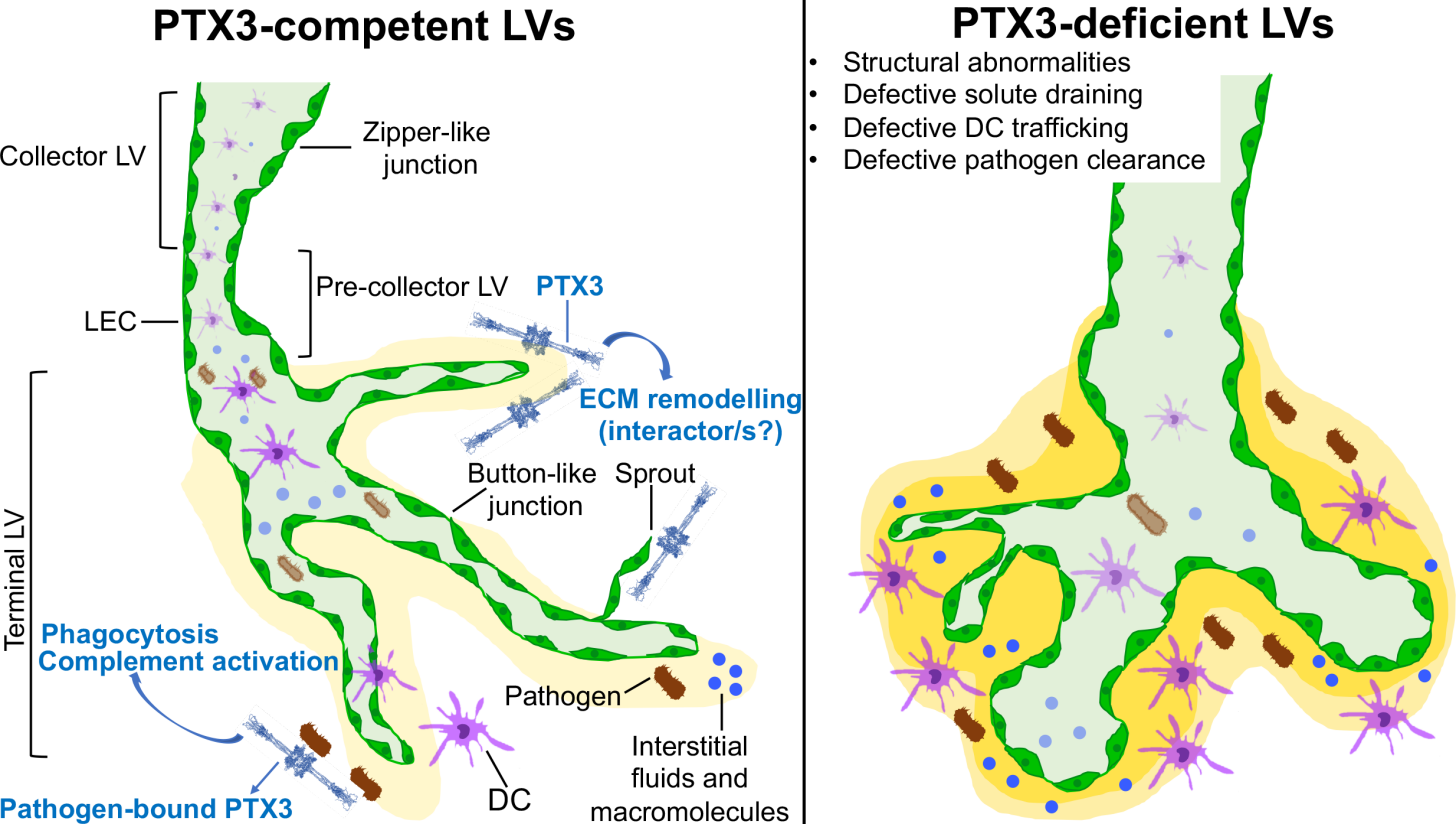
Schematic representation of PTX3 function in the organization and biology of LVs. PTX3 localizes in the ECM surrounding lymphatic terminals and LEC sprouting and is essential in the organization and function of the lymphatic system.

Stemming from these previous observations, the present study was designed to investigate the localization and function of PTX3 in the lymphatic system. In the diaphragm, trachea, intestine, and skin, organs extensively utilized for studies of lymphatic organization and function (e.g., in development, inflammation, and cancer) (61, 65, 66), PTX3 was localized in the ECM surrounding lymphatic terminals and LEC sprouting. General or LEC-restricted genetic deficiency resulted in abnormalities in the lymphatic system, including disorganization and hyperplasia of initial LV capillaries and alterations of tight junction-associated molecules. Thus, PTX3 is localized in the ECM of specialized terminal portions of the lymphatic system and is essential for its organization and function (**Figure 8**).

In the diaphragm tendon, PTX3 was selectively localized in LVs and lacunae of the tendon but not in the peripheral muscle. LVs in these locations fulfill different functions in fluid drainage (36, 62). Moreover, in dermal terminal LVs (58) and medullary sinus LN (8, 12, 59), PTX3 expression was prominent in LEC clusters with distinct functional profiles. The actual role of PTX3 in specialized LVs or LEC clusters remains to be fully defined.

PTX3 interacts with components of the ECM and plays a role in tissues under conditions of homeostasis or tissue repair (49–52). In the cumulus oophorous, PTX3 interacts with TSG-6 and is essential for the organization of an HA-rich viscoelastic matrix, crucial for female fertility (51, 52). PTX3 interacts with fibrinogen/fibrin and plasminogen, and this tripartite interaction facilitates the removal of the provisional fibrin matrix and wound healing (49). We failed to observe colocalization of PTX3 with TSG-6 in LV (**Supplementary Figure S7**). Emilin-1 is an important component of the LV ECM and was found to be co-expressed in PTX3-LEC (58). Emilin-1-deficient LVs showed abnormalities reminiscent of those reported here (63, 72). In whole-mount microscopy analyses of mouse colon, colocalization of PTX3 and Emilin-1 was selectively observed around terminal LVs projecting toward muscularis mucosa from submucosa to lamina propria (**Supplementary Figure S8A**). This was not the case in the diaphragm, where Emilin-1 localized scattered in the mesothelial cell layer (**Supplementary Figure S8B**). We did not observe *in vitro* binding of PTX3 to Emilin-1 (**Supplementary Figure S8C**). Thus, although the results reported here show that PTX3 is localized in the ECM in selected parts of the lymphatic system, its potential matrix partners remain to be defined.

While DC migration to the LN is reported to peak within 24 hours and continues for longer times in skin inflammation (73), in PTX3-deficient mice, we observed defective recruitment to the LN at 6 h and only a tendency at 12 h. It is known that LYVE-1 expressed on the basolateral surface of terminal LECs contributes to the early migration of DCs through LVs (74). In particular, the efficient trafficking of DCs from the skin to LVs relies on the interaction between LYVE-1 expressed on LECs and HA in the ECM, which coats DCs and allows docking to the lymphatic endothelium, facilitating their passage into the vessel lumen (74). In general, the interaction between ECM constituents and LECs is crucial for lymphangiogenesis and LV function (75). Therefore, the ability of PTX3 to orchestrate and remodel ECM (49, 52, 76) may explain the differences in ECM composition associated with reduction of HA in the non-fibrillar ECM of the pericellular spaces in LVs, underlying the functional defects associated with LVs in PTX3 deficiency. In addition, while recent lines of evidence point to a role of soluble and membrane-associated mediators such as CXCL12, CXCL1 and VCAM-1 on the regulation of DC migration to the LN through lymphatic collectors (77), PTX3 localized around lymphatic capillaries could facilitate DC migration at early time points, as observed in our setting. The altered organization of LEC junctions in terminal LVs observed in PTX3 deficiency and the role of the ECM deserves further studies.

PTX3 is a component of humoral innate immunity with antibody-like properties (40, 44, 69, 78, 79). Moreover, under selected conditions, this “ante-antibody” has been reported to contribute to adaptive immune responses (80, 81). Appropriate shaping and organization of LVs will likely be permissive for full activation of adaptive responses. Moreover, being strategically located at LV terminals, PTX3 may serve as a gatekeeper and facilitator of subsequent steps in pathogen handling. The higher number of *Salmonella* observed in mesenteric LNs of *Ptx3*-deficient mice after oral challenge may reflect both the antibody-like function of PTX3 and the altered LV organization.

The results reported here show that PTX3, produced by LEC, is selectively localized in the ECM surrounding lymphatic terminals and sprouting, and contributes to their structural organization and immunological function (**Figure 8**). The functional significance of PTX3 expression in specialized LEC clusters such as PTX3-LEC in LNs remains to be defined.

## Data Availability

The raw data supporting the conclusions of this article will be made available by the authors, without undue reservation.

## References

[1] Oliver G Kipnis J Randolph GJ Harvey NL. The lymphatic vasculature in the 21(st) century: novel functional roles in homeostasis and disease. Cell. (2020) 182:270–96. doi: 10.1016/j.cell.2020.06.039 PMC7392116 32707093

[2] Angeli V Lim HY. Biomechanical control of lymphatic vessel physiology and functions. Cell Mol Immunol. (2023) 20:1051–62. doi: 10.1038/s41423-023-01042-9 PMC10469203 37264249

[3] Maisel K Sasso MS Potin L Swartz MA. Exploiting lymphatic vessels for immunomodulation: Rationale, opportunities, and challenges. Adv Drug Delivery Rev. (2017) 114:43–59. doi: 10.1016/j.addr.2017.07.005 PMC6026542 28694027

[4] Fujimoto N He Y D’Addio M Tacconi C Detmar M Dieterich LC. Single-cell mapping reveals new markers and functions of lymphatic endothelial cells in lymph nodes. PloS Biol. (2020) 18:e3000704. doi: 10.1371/journal.pbio.3000704 32251437 PMC7162550

[5] Jalkanen S Salmi M. Lymphatic endothelial cells of the lymph node. Nat Rev Immunol. (2020) 20:566–78. doi: 10.1038/s41577-020-0281-x 32094869

[6] Park SM Angel CE McIntosh JD Mansell C Chen CJ Cebon J. Mapping the distinctive populations of lymphatic endothelial cells in different zones of human lymph nodes. PloS One. (2014) 9:e94781. doi: 10.1371/journal.pone.0094781 24733110 PMC3986404

[7] Takeda A Hollmen M Dermadi D Pan J Brulois KF Kaukonen R. Single-cell survey of human lymphatics unveils marked endothelial cell heterogeneity and mechanisms of homing for neutrophils. Immunity. (2019) 51:561–72 e5. doi: 10.1016/j.immuni.2019.06.027 31402260

[8] Xiang M Grosso RA Takeda A Pan J Bekkhus T Brulois K. A single-cell transcriptional roadmap of the mouse and human lymph node lymphatic vasculature. Front Cardiovasc Med. (2020) 7:52. doi: 10.3389/fcvm.2020.00052 32426372 PMC7204639

[9] Girard JP Moussion C Forster R. HEVs, lymphatics and homeostatic immune cell trafficking in lymph nodes. Nat Rev Immunol. (2012) 12:762–73. doi: 10.1038/nri3298 23018291

[10] Malhotra D Fletcher AL Astarita J Lukacs-Kornek V Tayalia P Gonzalez SF. Transcriptional profiling of stroma from inflamed and resting lymph nodes defines immunological hallmarks. Nat Immunol. (2012) 13:499–510. doi: 10.1038/ni.2262 22466668 PMC3366863

[11] Randolph GJ Ivanov S Zinselmeyer BH Scallan JP. The lymphatic system: integral roles in immunity. Annu Rev Immunol. (2017) 35:31–52. doi: 10.1146/annurev-immunol-041015-055354 27860528 PMC5551392

[12] Takeda A Salmi M Jalkanen S. Lymph node lymphatic endothelial cells as multifaceted gatekeepers in the immune system. Trends Immunol. (2023) 44:72–86. doi: 10.1016/j.it.2022.10.010 36463086

[13] Farnsworth RH Karnezis T Maciburko SJ Mueller SN Stacker SA. The interplay between lymphatic vessels and chemokines. Front Immunol. (2019) 10:518. doi: 10.3389/fimmu.2019.00518 31105685 PMC6499173

[14] Iftakhar EKI Fair-Mäkelä R Kukkonen-Macchi A Elima K Karikoski M Rantakari P. Gene-expression profiling of different arms of lymphatic vasculature identifies candidates for manipulation of cell traffic. Proc Natl Acad Sci U.S.A. (2016) 113:10643–8. doi: 10.1073/pnas.1602357113 PMC5035878 27601677

[15] Vigl B Aebischer D Nitschké M Iolyeva M Röthlin T Antsiferova O. Tissue inflammation modulates gene expression of lymphatic endothelial cells and dendritic cell migration in a stimulus-dependent manner. Blood. (2011) 118:205–15. doi: 10.1182/blood-2010-12-326447 21596851

[16] Nibbs RJ Kriehuber E Ponath PD Parent D Qin S Campbell JD. The beta-chemokine receptor D6 is expressed by lymphatic endothelium and a subset of vascular tumors. Am J Pathol. (2001) 158:867–77. doi: 10.1016/s0002-9440(10)64035-7 PMC1850343 11238036

[17] Savino B Caronni N Anselmo A Pasqualini F Borroni EM Basso G. ERK-dependent downregulation of the atypical chemokine receptor D6 drives tumor aggressiveness in Kaposi sarcoma. Cancer Immunol Res. (2014) 2:679–89. doi: 10.1158/2326-6066.CIR-13-0202 24844911

[18] Cyster JG Schwab SR. Sphingosine-1-phosphate and lymphocyte egress from lymphoid organs. Annu Rev Immunol. (2012) 30:69–94. doi: 10.1146/annurev-immunol-020711-075011 22149932

[19] Johnson LA Jackson DG. Hyaluronan and its receptors: key mediators of immune cell entry and trafficking in the lymphatic system. Cells. (2021) 10(8):2061. doi: 10.3390/cells10082061 PMC8393520 34440831

[20] Schineis P Runge P Halin C. Cellular traffic through afferent lymphatic vessels. Vascul Pharmacol. (2019) 112:31–41. doi: 10.1016/j.vph.2018.08.001 30092362

[21] Ulvmar MH Werth K Braun A Kelay P Hub E Eller K. The atypical chemokine receptor CCRL1 shapes functional CCL21 gradients in lymph nodes. Nat Immunol. (2014) 15:623–30. doi: 10.1038/ni.2889 24813163

[22] Moran I Grootveld AK Nguyen A Phan TG. Subcapsular sinus macrophages: the seat of innate and adaptive memory in murine lymph nodes. Trends Immunol. (2019) 40:35–48. doi: 10.1016/j.it.2018.11.004 30502023

[23] Zhang W Li J Liang J Qi X Tian J Liu J. Coagulation in lymphatic system. Front Cardiovasc Med. (2021) 8:762648. doi: 10.3389/fcvm.2021.762648 34901222 PMC8652051

[24] Liao S von der Weid PY. Lymphatic system: an active pathway for immune protection. Semin Cell Dev Biol. (2015) 38:83–9. doi: 10.1016/j.semcdb.2014.11.012 PMC4397130 25534659

[25] Dieterich LC Tacconi C Ducoli L Detmar M. Lymphatic vessels in cancer. Physiol Rev. (2022) 102:1837–79. doi: 10.1152/physrev.00039.2021 35771983

[26] Karpanen T Egeblad M Karkkainen MJ Kubo H Yla-Herttuala S Jaattela M. Vascular endothelial growth factor C promotes tumor lymphangiogenesis and intralymphatic tumor growth. Cancer Res. (2001) 61:1786–90. 11280723

[27] Mandriota SJ Jussila L Jeltsch M Compagni A Baetens D Prevo R. Vascular endothelial growth factor-C-mediated lymphangiogenesis promotes tumour metastasis. EMBO J. (2001) 20:672–82. doi: 10.1093/emboj/20.4.672 PMC145430 11179212

[28] Norden PR Kume T. Molecular mechanisms controlling lymphatic endothelial junction integrity. Front Cell Dev Biol. (2021) 8:627647. doi: 10.3389/fcell.2020.627647 33521001 PMC7841202

[29] Sutherland TE Dyer DP Allen JE. The extracellular matrix and the immune system: A mutually dependent relationship. Science. (2023) 379:eabp8964. doi: 10.1126/science.abp8964 36795835

[30] Wiig H Keskin D Kalluri R. Interaction between the extracellular matrix and lymphatics: consequences for lymphangiogenesis and lymphatic function. Matrix Biol. (2010) 29:645–56. doi: 10.1016/j.matbio.2010.08.001 PMC3992865 20727409

[31] Czarnowska E Ratajska A Jankowska-Steifer E Flaht-Zabost A Niderla-Bielinska J. Extracellular matrix molecules associated with lymphatic vessels in health and disease. Histol Histopathol. (2024) 39:13–34. doi: 10.14670/HH-18-641 37350542

[32] Baluk P Fuxe J Hashizume H Romano T Lashnits E Butz S. Functionally specialized junctions between endothelial cells of lymphatic vessels. J Exp Med. (2007) 204:2349–62. doi: 10.1084/jem.20062596 PMC2118470 17846148

[33] Baluk P McDonald DM. Buttons and zippers: endothelial junctions in lymphatic vessels. Cold Spring Harb Perspect Med. (2022) 12(12):a04117. doi: 10.1101/cshperspect.a041178 PMC9643678 35534209

[34] Negrini D Moriondo A. Pleural function and lymphatics. Acta Physiol (Oxf). (2013) 207:244–59. doi: 10.1111/apha.12016 23009260

[35] Achen MG McColl BK Stacker SA. Focus on lymphangiogenesis in tumor metastasis. Cancer Cell. (2005) 7:121–7. doi: 10.1016/j.ccr.2005.01.017 15710325

[36] Hahn C Schwartz MA. Mechanotransduction in vascular physiology and atherogenesis. Nat Rev Mol Cell Biol. (2009) 10:53–62. doi: 10.1038/nrm2596 19197332 PMC2719300

[37] Avraamides CJ Garmy-Susini B Varner JA. Integrins in angiogenesis and lymphangiogenesis. Nat Rev Cancer. (2008) 8:604–17. doi: 10.1038/nrc2353 PMC2577722 18497750

[38] Janssen L Dupont L Bekhouche M Noel A Leduc C Voz M. ADAMTS3 activity is mandatory for embryonic lymphangiogenesis and regulates placental angiogenesis. Angiogenesis. (2016) 19:53–65. doi: 10.1007/s10456-015-9488-z 26446156 PMC4700087

[39] Rundhaug JE. Matrix metalloproteinases and angiogenesis. J Cell Mol Med. (2005) 9:267–85. doi: 10.1111/j.1582-4934.2005.tb00355.x PMC6740080 15963249

[40] Garlanda C Hirsch E Bozza S Salustri A De Acetis M Nota R. Non-redundant role of the long pentraxin PTX3 in anti-fungal innate immune response. Nature. (2002) 420:182–6. doi: 10.1038/nature01195 12432394

[41] Mantovani A Garlanda C. Humoral innate immunity and acute-phase proteins. N Engl J Med. (2023) 388:439–52. doi: 10.1056/NEJMra2206346 PMC9912245 36724330

[42] Bottazzi B Doni A Garlanda C Mantovani A. An integrated view of humoral innate immunity: pentraxins as a paradigm. Annu Rev Immunol. (2010) 28:157–83. doi: 10.1146/annurev-immunol-030409-101305 19968561

[43] He Q Li H Rui Y Liu L He B Shi Y. Pentraxin 3 gene polymorphisms and pulmonary aspergillosis in chronic obstructive pulmonary disease patients. Clin Infect Dis. (2018) 66:261–7. doi: 10.1093/cid/cix749 29020397

[44] Jeannin P Bottazzi B Sironi M Doni A Rusnati M Presta M. Complexity and complementarity of outer membrane protein A recognition by cellular and humoral innate immunity receptors. Immunity. (2005) 22:551–60. doi: 10.1016/j.immuni.2005.03.008 15894273

[45] Lu J Marnell LL Marjon KD Mold C Du Clos TW Sun PD. Structural recognition and functional activation of FcgammaR by innate pentraxins. Nature. (2008) 456:989–92. doi: 10.1038/nature07468 PMC2688732 19011614

[46] Turati M Giacomini A Rezzola S Maccarinelli F Gazzaroli G Valentino S. The natural FGF-trap long pentraxin 3 inhibits lymphangiogenesis and lymphatic dissemination. Exp Hematol Oncol. (2022) 11:84. doi: 10.1186/s40164-022-00330-w 36320051 PMC9623950

[47] Porte R Silva-Gomes R Theroude C Parente R Asgari F Sironi M. Regulation of inflammation and protection against invasive pneumococcal infection by the long pentraxin PTX3. Elife. (2023) 12:1-29. doi: 10.7554/eLife.78601 PMC10266767 37222419

[48] Garlanda C Bottazzi B Magrini E Inforzato A Mantovani A. PTX3, a humoral pattern recognition molecule, in innate immunity, tissue repair, and cancer. Physiol Rev. (2018) 98:623–39. doi: 10.1152/physrev.00016.2017 PMC5985957 29412047

[49] Doni A Musso T Morone D Bastone A Zambelli V Sironi M. An acidic microenvironment sets the humoral pattern recognition molecule PTX3 in a tissue repair mode. J Exp Med. (2015) 212:905–25. doi: 10.1084/jem.20141268 PMC4451130 25964372

[50] Grcevic D Sironi M Valentino S Deban L Cvija H Inforzato A. The long pentraxin 3 plays a role in bone turnover and repair. Front Immunol. (2018) 9:417. doi: 10.3389/fimmu.2018.00417 29556234 PMC5845433

[51] Salustri A Garlanda C Hirsch E De Acetis M Maccagno A Bottazzi B. PTX3 plays a key role in the organization of the cumulus oophorus extracellular matrix and in *in vivo* fertilization. Development. (2004) 131:1577–86. doi: 10.1242/dev.01056 14998931

[52] Scarchilli L Camaioni A Bottazzi B Negri V Doni A Deban L. PTX3 interacts with inter-alpha-trypsin inhibitor: implications for hyaluronan organization and cumulus oophorus expansion. J Biol Chem. (2007) 282:30161–70. doi: 10.1074/jbc.M703738200 17675295

[53] Bonacina F Barbieri SS Cutuli L Amadio P Doni A Sironi M. Vascular pentraxin 3 controls arterial thrombosis by targeting collagen and fibrinogen induced platelets aggregation. Biochim Biophys Acta. (2016) 1862:1182–90. doi: 10.1016/j.bbadis.2016.03.007 PMC4856734 26976330

[54] Cappuzzello C Doni A Dander E Pasqualini F Nebuloni M Bottazzi B. Mesenchymal stromal cell-derived PTX3 promotes wound healing via fibrin remodeling. J Invest Dermatol. (2016) 136:293–300. doi: 10.1038/JID.2015.346 26763449

[55] Amatschek S Kriehuber E Bauer W Reininger B Meraner P Wolpl A. Blood and lymphatic endothelial cell-specific differentiation programs are stringently controlled by the tissue environment. Blood. (2007) 109:4777–85. doi: 10.1182/blood-2006-10-053280 17289814

[56] Wick N Saharinen P Saharinen J Gurnhofer E Steiner CW Raab I. Transcriptomal comparison of human dermal lymphatic endothelial cells ex vivo and *in vitro* . Physiol Genomics. (2007) 28:179–92. doi: 10.1152/physiolgenomics.00037.2006 17234577

[57] Sironi M Conti A Bernasconi S Fra AM Pasqualini F Nebuloni M. Generation and characterization of a mouse lymphatic endothelial cell line. Cell Tissue Res. (2006) 325:91–100. doi: 10.1007/s00441-006-0171-y 16534603

[58] Petkova M Kraft M Stritt S Martinez-Corral I Ortsater H Vanlandewijck M. Immune-interacting lymphatic endothelial subtype at capillary terminals drives lymphatic malformation. J Exp Med. (2023) 220(4):e20220741. doi: 10.1084/jem.20220741 PMC9884640 36688917

[59] Abe Y Sakata-Yanagimoto M Fujisawa M Miyoshi H Suehara Y Hattori K. A single-cell atlas of non-haematopoietic cells in human lymph nodes and lymphoma reveals a landscape of stromal remodelling. Nat Cell Biol. (2022) 24:565–78. doi: 10.1038/s41556-022-00866-3 PMC9033586 35332263

[60] Bazigou E Lyons OT Smith A Venn GE Cope C Brown NA. Genes regulating lymphangiogenesis control venous valve formation and maintenance in mice. J Clin Invest. (2011) 121:2984–92. doi: 10.1172/JCI58050 PMC3223924 21765212

[61] Yao LC Baluk P Srinivasan RS Oliver G McDonald DM. Plasticity of button-like junctions in the endothelium of airway lymphatics in development and inflammation. Am J Pathol. (2012) 180:2561–75. doi: 10.1016/j.ajpath.2012.02.019 PMC3378913 22538088

[62] Li J Zhao Z Zhou J Yu S. A study of the three-dimensional organization of the human diaphragmatic lymphatic lacunae and lymphatic drainage units. Ann Anat. (1996) 178:537–44. doi: 10.1016/S0940-9602(96)80113-0 9010570

[63] Danussi C Spessotto P Petrucco A Wassermann B Sabatelli P Montesi M. Emilin1 deficiency causes structural and functional defects of lymphatic vasculature. Mol Cell Biol. (2008) 28:4026–39. doi: 10.1128/MCB.02062-07 PMC2423131 18411305

[64] Spadoni I Zagato E Bertocchi A Paolinelli R Hot E Di Sabatino A. A gut-vascular barrier controls the systemic dissemination of bacteria. Science. (2015) 350:830–4. doi: 10.1126/science.aad0135 26564856

[65] Suh SH Choe K Hong SP Jeong SH Makinen T Kim KS. Gut microbiota regulates lacteal integrity by inducing VEGF-C in intestinal villus macrophages. EMBO Rep. (2019) 20(4):e46927. doi: 10.15252/embr.201846927 PMC6446200 30783017

[66] Yoshimatsu Y Lee YG Akatsu Y Taguchi L Suzuki HI Cunha SI. Bone morphogenetic protein-9 inhibits lymphatic vessel formation via activin receptor-like kinase 1 during development and cancer progression. Proc Natl Acad Sci U.S.A. (2013) 110:18940–5. doi: 10.1073/pnas.1310479110 PMC3839734 24133138

[67] Stritt S Koltowska K Makinen T. Homeostatic maintenance of the lymphatic vasculature. Trends Mol Med. (2021) 27:955–70. doi: 10.1016/j.molmed.2021.07.003 34332911

[68] Schwager S Detmar M. Inflammation and lymphatic function. Front Immunol. (2019) 10:308. doi: 10.3389/fimmu.2019.00308 30863410 PMC6399417

[69] Jaillon S Moalli F Ragnarsdottir B Bonavita E Puthia M Riva F. The humoral pattern recognition molecule PTX3 is a key component of innate immunity against urinary tract infection. Immunity. (2014) 40:621–32. doi: 10.1016/j.immuni.2014.02.015 24745336

[70] Asgari F Supino D Parente R Polentarutti N Stravalaci M Porte R. The long pentraxin PTX3 controls klebsiella pneumoniae severe infection. Front Immunol. (2021) 12:666198. doi: 10.3389/fimmu.2021.666198 34093560 PMC8173212

[71] Ohl ME Miller SI. Salmonella: a model for bacterial pathogenesis. Annu Rev Med. (2001) 52:259–74. doi: 10.1146/annurev.med.52.1.259 11160778

[72] Danussi C Del Bel Belluz L Pivetta E Modica TM Muro A Wassermann B. EMILIN1/alpha9beta1 integrin interaction is crucial in lymphatic valve formation and maintenance. Mol Cell Biol. (2013) 33:4381–94. doi: 10.1128/MCB.00872-13 PMC3838180 24019067

[73] Kissenpfennig A Henri S Dubois B Laplace-Builhé C Perrin P Romani N. Dynamics and function of Langerhans cells *in vivo*: dermal dendritic cells colonize lymph node areas distinct from slower migrating Langerhans cells. Immunity. (2005) 22:643–54. doi: 10.1016/j.immuni.2005.04.004 15894281

[74] Johnson LA Banerji S Lawrance W Gileadi U Prota G Holder KA. Dendritic cells enter lymph vessels by hyaluronan-mediated docking to the endothelial receptor LYVE-1. Nat Immunol. (2017) 18:762–70. doi: 10.1038/ni.3750 28504698

[75] Jayadev R Chi Q Keeley D Hastie E Kelley L Sherwood D. [amp]]alpha;-Integrins dictate distinct modes of type IV collagen recruitment to basement membranes. J Cell Biol. (2019) 218:jcb.201903124. doi: 10.1083/jcb.201903124 PMC6719451 31387941

[76] Rabelink TJ Wang G van der Vlag J van den Berg BM. The roles of hyaluronan in kidney development, physiology and disease. Nat Rev Nephrol. (2024) 20:822-32. doi: 10.1038/s41581-024-00883-5 39191935

[77] Arasa J Collado-Diaz V Kritikos I Medina-Sanchez JD Friess MC Sigmund EC. Upregulation of VCAM-1 in lymphatic collectors supports dendritic cell entry and rapid migration to lymph nodes in inflammation. J Exp Med. (2021) 218(7):e20201413. doi: 10.1084/jem.20201413 PMC8129804 33988714

[78] Bottazzi B Garlanda C Salvatori G Jeannin P Manfredi A Mantovani A. Pentraxins as a key component of innate immunity. Curr Opin Immunol. (2006) 18:10–5. doi: 10.1016/j.coi.2005.11.009 16343883

[79] Jaillon S Peri G Delneste Y Fremaux I Doni A Moalli F. The humoral pattern recognition receptor PTX3 is stored in neutrophil granules and localizes in extracellular traps. J Exp Med. (2007) 204:793–804. doi: 10.1084/jem.20061301 17389238 PMC2118544

[80] Bottazzi B Santini L Savino S Giuliani MM Duenas Diez AI Mancuso G. Recognition of Neisseria meningitidis by the long pentraxin PTX3 and its role as an endogenous adjuvant. PloS One. (2015) 10:e0120807. doi: 10.1371/journal.pone.0120807 25786110 PMC4364741

[81] Chorny A Casas-Recasens S Sintes J Shan M Polentarutti N Garcia-Escudero R. The soluble pattern recognition receptor PTX3 links humoral innate and adaptive immune responses by helping marginal zone B cells. J Exp Med. (2016) 213:2167–85. doi: 10.1084/jem.20150282 PMC5030794 27621420

