# Supplementary material for: PTX3 is expressed in terminal lymphatics and shapes their organization and function
Source: Front Immunol. 2024 Nov 21;15:1426869. doi: 10.3389/fimmu.2024.1426869 (PMC11617523; doi:10.3389/fimmu.2024.1426869)
Supplement: Supplementary file 1 [file DataSheet1.pdf]

Supplementary Material

PTX3 IS EXPRESSED IN TERMINAL LYMPHATICS AND SHAPES THEIR ORGANIZATION AND FUNCTION

Andrea Doni [1, †, *], Marina Sironi [2, †], Annalisa Del Prete [2, 3], Fabio Pasqualini [2], Sonia Valentino [2], Ivan Cuccovillo [2, ^], Raffaella Parente [1], Michela Calvi [4], Antonella Tosoni [5], Gianluca Vago [5, #], Manuela Nebuloni [5], Cecilia Garlanda [6, 7], Annunciata Vecchi [2], Barbara Bottazzi [2, **], Alberto Mantovani [2, 7, 8, *]**

[1] Unit of Multiscale and Nanostructural Imaging, IRCCS Humanitas Research Hospital, Milan, Italy
[2] Cellular and Humoral Innate Immunity Lab, IRCCS Humanitas Research Hospital, Milan, Italy
[3] Department of Molecular and Translational Medicine, University of Brescia, Brescia, Italy
[4] Clinical and Experimental Immunology Lab, IRCCS Humanitas Research Hospital, Milan, Italy
[5] Pathology Unit, L. Sacco Hospital, Department of Biomedical and Clinical Sciences, University of Milan, Milan, Italy.
[6], Experimental Immunopathology Lab, IRCCS Humanitas Research Hospital, Milan, Italy
[7] Department of Biomedical Sciences, Humanitas University, Milan, Italy
[8] William Harvey Research Institute, Queen Mary University of London, London, United Kingdom

Correspondence: [*], andrea.doni@humanitasresearch.it;
[**], barbara.bottazzi@humanitasresearch.it; [***], alberto.mantovani@humanitasresearch.it.

[†] These authors contributed equally to this work

[^] Present address: San Raffaele Telethon Institute for Gene Therapy (SR-TIGET), IRCCS Ospedale San Raffaele, Milan, Italy.
[#] Present address: Department of Oncology & Haemato-Oncology, University of Milan, Milan, Italy

Supplementary Data:
Supplementary Figures S1–S8 and related legends;
Legends of Videos S1-S5.

Supplementary Figures

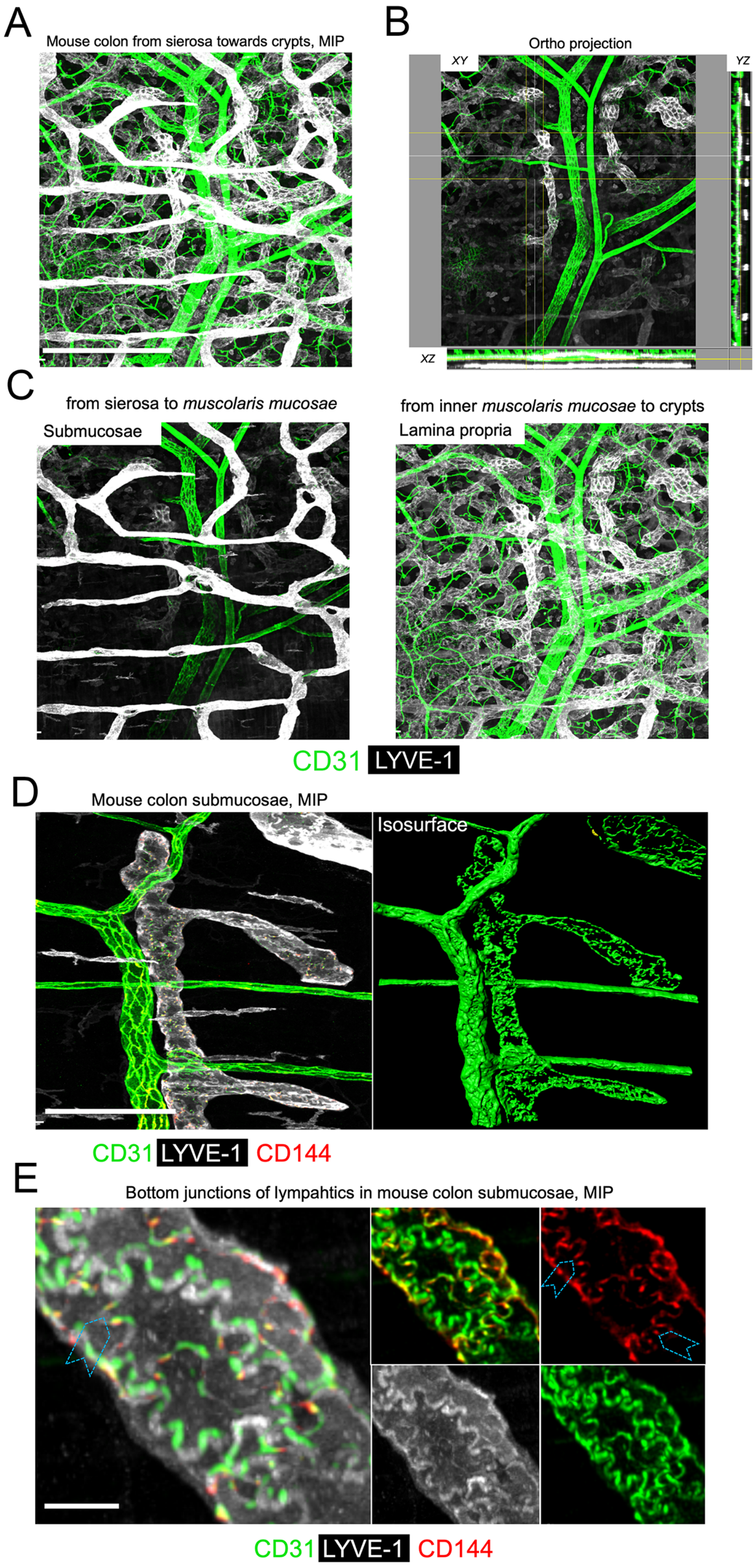

Figure S1. 3D reconstruction of lymphatic vasculature and visualization of LEC junctions. (**A**) representative maximum intensity projection (MIP) image of a *z*-stack obtained with 10X objective lens of mouse colon from sierosa towards crypts showing blood ($CD31^+$, green) and lymphatic vasculature (LYVE-1^+, white). Scale bar=500µm. (**B**) 3D visualization of (**A**) as *xz* and *yz* orthogonal planes. (**C**) MIP images after 3D rendering by separating lymphatic vasculature of the submucosa (left) from that of the *laminae* (right) of (**A**). (**D**) representative 3D isosurface reconstruction starting from fluorescence signal of *z*-stack MIP images obtained with 40X objective lens of mouse colon submucosa (left; $CD31^+$, green; LYVE-1^+, white). Scale=50µm. (**E**) representative 3D isosurface reconstruction from fluorescence signal of *z*-stack MIP images obtained with 60X objective lens of mouse colon submucosa showing junctions on single LEC (left; CD31, green; LYVE-1, white; CD144, red). Left panel, merge of fluorescence signal contributions. Right panel, extracted images of each fluorescence signal contribution. Blue arrowheads, representative button junctions. Scale bar=5µm.

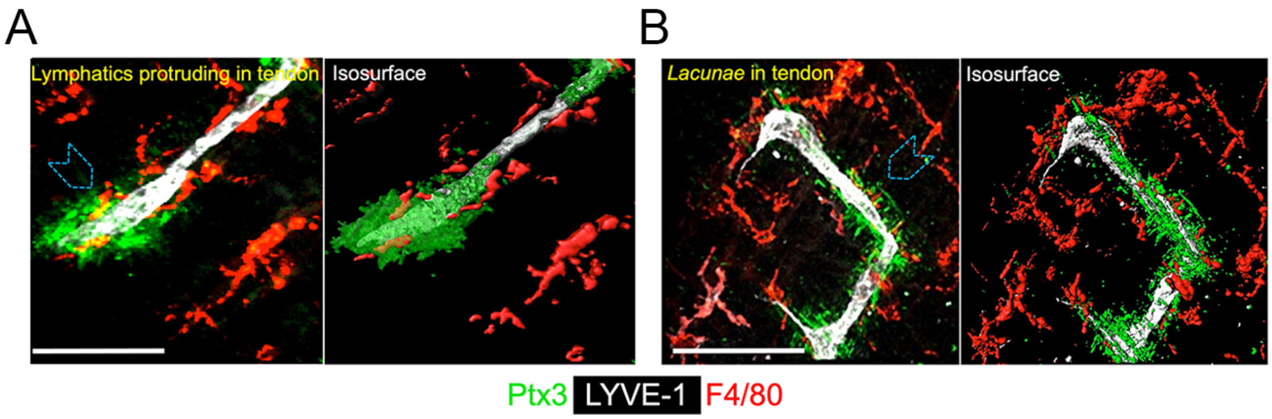

Figure S2. Localization of PTX3 around terminal lymphatic tips and in *lacunae* enriched of resident macrophages. Mouse diaphragm stained for LYVE-1 (white), F4/80 (red) and PTX3 (green). (**A**) MIP of *z*-stack confocal images of mouse tendon diaphragm showing LVs protruding from thoracic wall to central tendon. (**B**) MIP of *z*-stack confocal images of mouse tendon diaphragm showing LV *lacunae*. (**A** and **B**) blue arrowheads, PTX3 localization in ECM around blind ends of LVs associated with accumulation of F4/80^{+} tissue macrophages (red). Corresponding 3D isosurface rendering (right) is also shown. Scale bar=10µm.

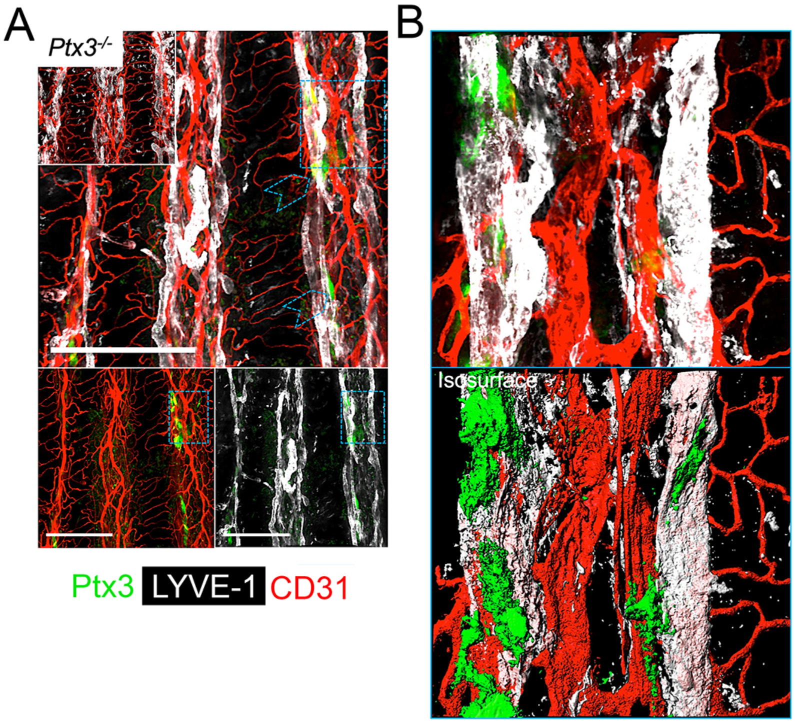

Figure S3. Localization of PTX3 at lymphatic vessels in mouse trachea. (**A**) upper panel, MIP of *z*-stack images of $CD31^+$ blood (red) and LYVE-1^+ LVs (white) and PTX3 localization (green). Lower panel, signal contribution of CD31 and PTX3 (left) or LYVE-1 and PTX3 (right). Blue arrowheads, PTX3 around blind-ended rounded initial LVs. Inset, *Ptx3*$^{-/-}$ trachea as control. Scale bar=500µm. (**B**) upper panel, close-up image of the blue dotted line area in (**A**) showing blind-ended terminals in colon trachea. Lower panel, 3D rendering of fluorescence signals in the upper panel showed as isosurface creation.

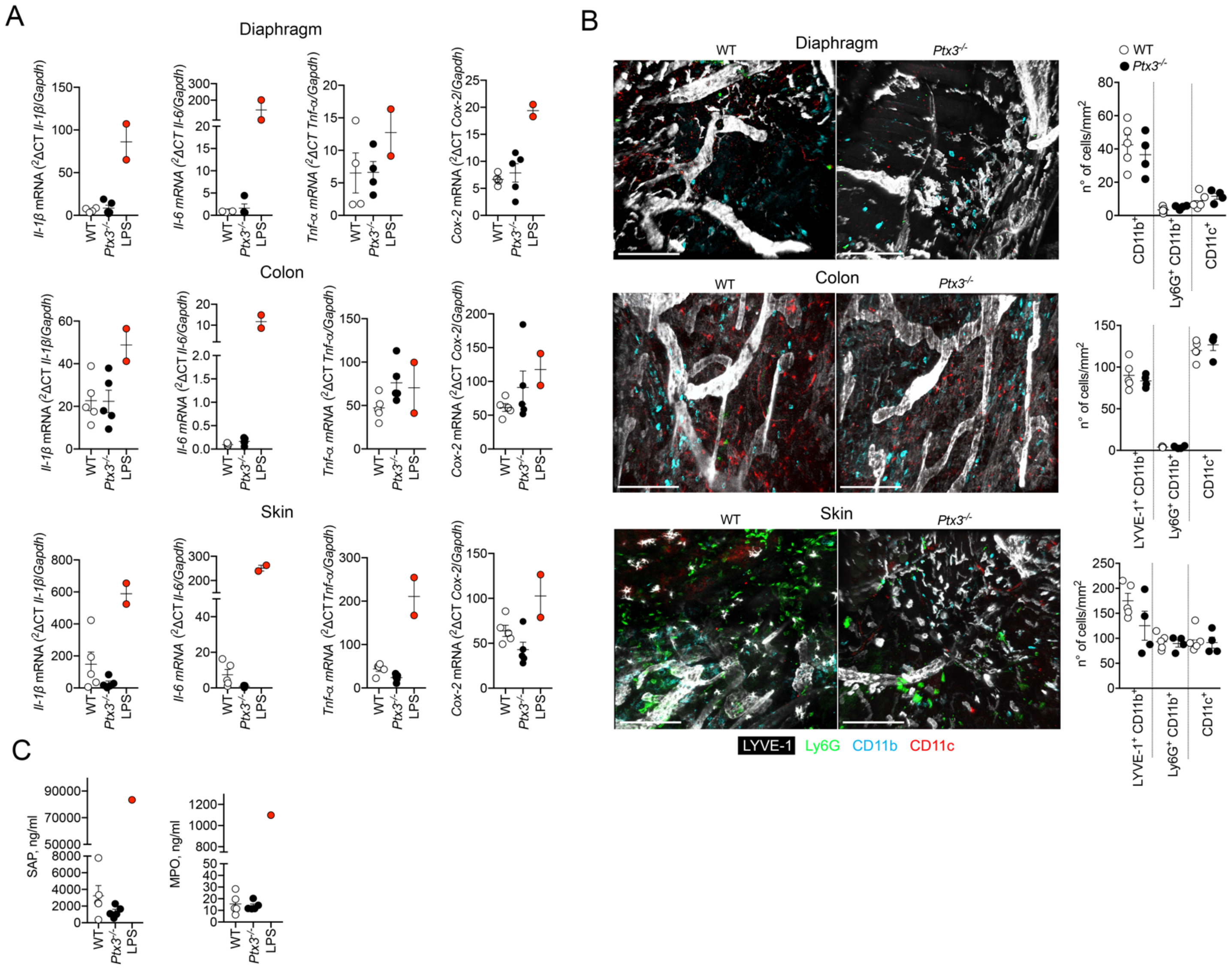

Figure S4. Levels of inflammatory mediators and leukocytes in control and PTX3 deficient mice in resting conditions. (**A**) real-time PCR analyses for *Il-1β*, *Il-6, Tnf-α* and *Cox-2* performed on extracted RNA from homogenized diaphragm (upper), colon (middle) and ear skin (lower) of WT (n=5) and *Ptx3*$^{-/-}$ (n=5) mice. LPS (2µg/mouse) was injected i.p. in WT mice (n=2) 16h before organ collection and used as control. As reported in Material and Methods section, ROUT method (Q=1%) was used to identify outliers in the data groups. Each spot refers to a single mouse and expression of the averaged absolute value ±SEM of a triplicate analyzed with the Δ^2CT method and normalized on GAPDH expression. (**B**) analysis of abundance of leukocytes in proximity of LVs in the diaphragm (upper), colon (middle) and ear skin (lower) of WT (n=5) and *Ptx3*$^{-/-}$ (n=5) mice. Left, representative MIP confocal images obtained from *z*-stacks (0.75µm/slice; 1024x1024 pixels) of the entire organ out of n=2-7 (diaphragm), n=2-10 (colon), n=4-8 (ear skin) acquired per genotype. White, LYVE-1; green, Ly6G; cyan, CD11b; red, CD11c. Scale bar=100µm. Right, quantification of leucocytes in the tissues as reported in Material and Methods section after 3D reconstruction of the *z*-stacks. Each spot corresponds to mean of sum of number of spots (single cells) ± SEM counted automatically in n=2-7

(diaphragm), n=2-10 (colon), n=4-8 (ear skin) MIP images per genotype. Results are expression of the number of LYVE-1$^+$ CD11b$^+$ resident macrophages (colon, skin), Ly6G$^-$ CD11b$^+$ mono-macrophages (diaphragm), Ly6G$^+$ CD11b$^+$ neutrophils (colon, diaphragm, skin) and CD11c$^+$ DCs (colon, diaphragm, skin) per mm^2 of tissue area. (**C**) ELISA for SAP and MPO in ACD-plasma of WT (n=4) and *Ptx3*$^{-/-}$ (n=4) mice. LPS (2µg/mouse) was injected i.p. in a WT mouse and used as control. Each spot corresponds to a single mouse. Results are mean ± SEM of a triplicate.

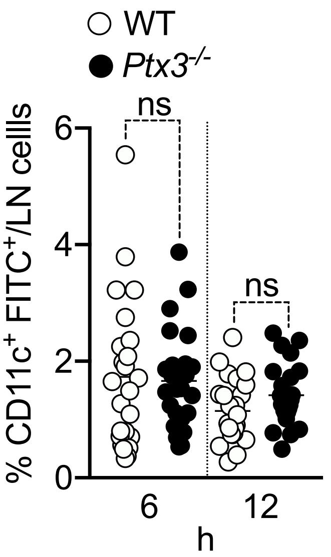

Figure S5. DC trafficking show as frequency of FITC^{+}CD11c^{+} DCs amongst all LN cells. Each spot corresponds to a single LN.

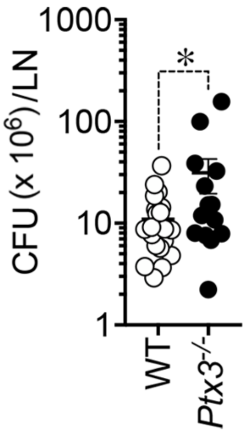

Figure S6. Dissemination of *Salmonella enterica* via the lymphatic system. The ΔaroA auxotrophic *Salmonella enterica* serovar typhimurium mutant (10^9) was administrated via *os*. Number of bacteria was counted (24h) in the mesenteric LNs and was 10.97±1.67x10^6/LN in WT vs 31.22±11.73x10^6/LN in *Ptx3*$^{-/-}$). Each spot corresponds to a single LN (WT, n=23; *Ptx3*$^{-/-}$, n=14). 2 pooled experiments performed on C57BL/6J genetic background mice. *, P<0.05 (two-tailed; unpaired, t test).

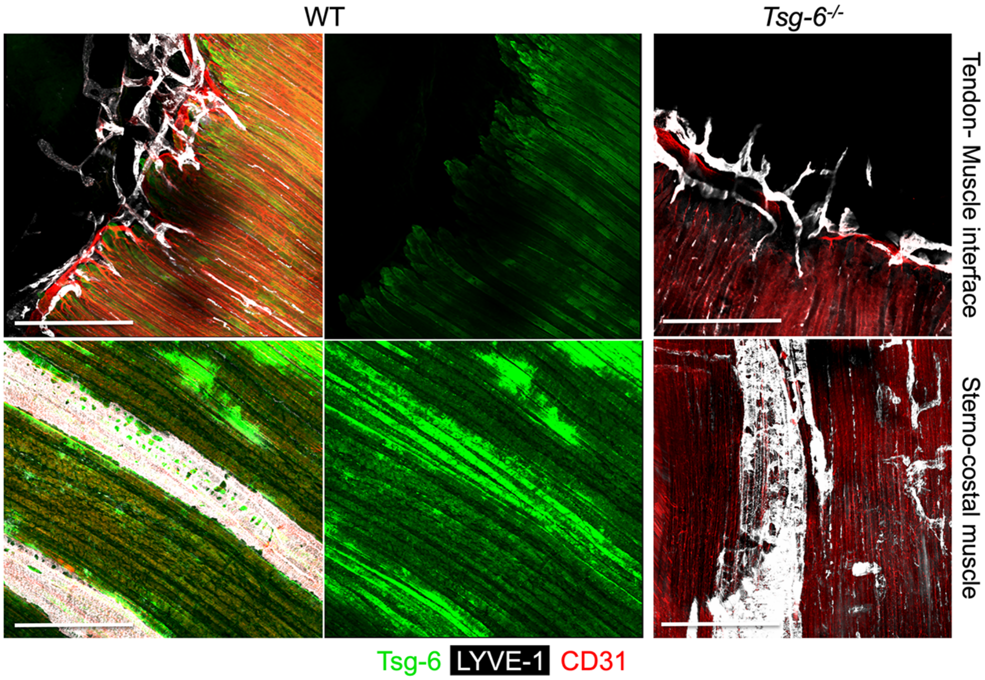

Figure S7. Localization of TSG-6 in lymphatic vessels in the mouse diaphragm. MIP images of mouse diaphragm stained for LYVE-1 (white), CD31 (red) and TSG-6 (green) showing negative staining for TSG-6 in LVs protruding from muscle to tendon (upper panels), but TSG-6 positivity associated around LVs at sterno-costal muscle (lower panels). *Tsg-6*$^{-/-}$ animals (right panels) were used as control. Scale bar=500µm (upper panels) and 50µm (lower panels).

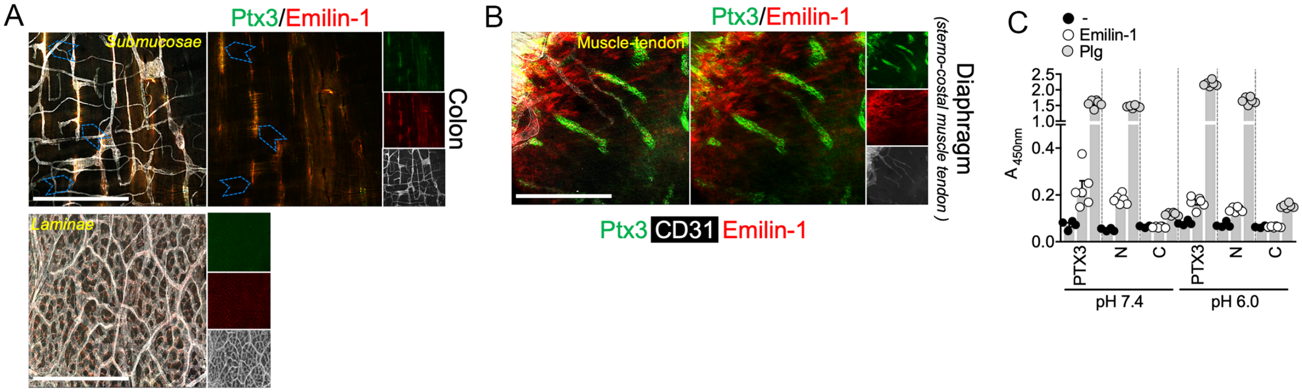

Figure S8. Colocalization of PTX3 with potential interactors of ECM at initial lymphatics. (**A**) MIP images of submucosa (upper panel) and lamina (lower panel) of a mouse colon stained for CD31 (white), PTX3 (green) and Emilin-1 (red). Blue arrowheads in the upper panels indicate PTX3 colocalization with Emilin-1 at ECM of blind-ended initial LVs protruding from sierosa toward *muscolaris* mucosa. On the right are shown the MIP images of single signal contributions of PTX3 (upper), Emilin-1 (middle) and Lyve-1 (lower). (**B**) MIP images of mouse diaphragm stained for CD31 (white), PTX3 (green) and Emilin-1 (red). Middle extracted image of signal contribution of PTX3 and Emilin-1. Scale bar=500µm. (**C**) Binding of PTX3 (0.2µg/ml) or N and C terminal domain (0.2µg/ml) to recombinant Emilin-1 fragment (aa 676≈1016; gC1q domain; 0.5µg/well) or native purified plasminogen (0.5µg/well) at different pHs by ELISA. Mean±SE of triplicate O.D. (A_{450nm}) of 2 experiments performed and pooled.

Supplementary Data

Videos S1 and **S2**. Method for 3D analysis of LVs using confocal microscopy. Examples of *z*-stacks of mouse colon and 3D isosurface reconstruction related to Supplementary Figure 1 F-H. *Z*-stacks of mouse colon obtained with 10X (**Video S1**) (n=1-6 *z*-stacks; n= 4 WT mice) or 40X (**Video S2**) (n=2-7 *z*-stacks; n= 4 WT mice) objective lens from sierosa towards crypts showing blood ($CD31^{+}$, green) and lymphatic vasculature (LYVE-1^{+}, white). The scale bars refer to Supplementary Figure 1A (Video S1) or Supplementary Figure 1D (Video S2).

Video S3. *z*-stack of mouse diaphragm and 3D isosurface reconstruction. **Videos S3** is related to Figures 1A and shows the localization of PTX3 (green) around LVs (LYVE-1^{+}, white) of diaphragm at muscle-tendon interface (n=2-4 *z*-stacks; n= 2 WT mice). The blood vessel bordering the muscle and tendon portion was negative for PTX3. Red, α-SMA^{+} pericytes. Scale bar at starting frame= 150μm.

Video S4. *z*-stack of mouse diaphragm and 3D isosurface reconstruction. **Video S4** is related to Figures 1E and shows PTX3 (green) localization around LEC sprouting in mouse diaphragm tendon. White, LYVE-1. Scale bar at starting frame= 70μm

Video S5. 3D isosurface reconstruction out of n=3 and 4 *z*-stacks per n= 2 WT mice acquired showing PTX3 localization around LV terminals enriched of tissue macrophages in diaphragm tendon**. Video S5 is** related to Supplementary Figure 2. Green, PTX3; red, F4/80; LYVE-1, white. Scale bar at starting frame= 80μm.
